# Disruption of MAM integrity in mutant *FUS* oligodendroglial progenitors from hiPSCs

**DOI:** 10.1007/s00401-023-02666-x

**Published:** 2024-01-03

**Authors:** Yingli Zhu, Thibaut Burg, Katrien Neyrinck, Tim Vervliet, Fatemeharefeh Nami, Ellen Vervoort, Karan Ahuja, Maria Livia Sassano, Yoke Chin Chai, Arun Kumar Tharkeshwar, Jonathan De Smedt, Haibo Hu, Geert Bultynck, Patrizia Agostinis, Johannes V. Swinnen, Ludo Van Den Bosch, Rodrigo Furtado Madeiro da Costa, Catherine Verfaillie

**Affiliations:** 1 Department of Development and Regeneration, Stem Cell Institute, KU Leuven, 3000 Leuven, Belgium; 2 Department of Neurosciences, Experimental Neurology, KU Leuven, Leuven Brain Institute (LBI), 3000 Leuven, Belgium; 3 Laboratory of Neurobiology, VIB, Center for Brain and Disease Research, 3000 Leuven, Belgium; 4 Laboratory of Molecular and Cellular Signalling, Department of Cellular and Molecular Medicine, KU Leuven, 3000 Leuven, Belgium; 5 Laboratory of Cell Death Research and Therapy, Department of Cellular and Molecular Medicine, KU Leuven, 3000 Leuven, Belgium; 6 Center for Cancer Biology, VIB, 3000 Leuven, Belgium; 7 Animal Physiology and Neurobiology Section, Department of Biology, Neural Circuit Development and Regeneration Research Group, 3000 Leuven, Belgium; 8 National Engineering Research Center for Modernization of Traditional Chinese Medicine-Hakka Medical Resources Branch, School of Pharmacy, Gannan Medical University, Ganzhou, China; 9 Laboratory of Lipid Metabolism and Cancer, Department of Oncology, KU Leuven, 3000 Leuven, Belgium

**Keywords:** Amyotrophic lateral sclerosis, FUS, Lipid defects, MAM disruption, ER stress, Mitochondrial dysfunction

## Abstract

Amyotrophic lateral sclerosis (ALS) is a rapidly progressive and fatal neurodegenerative disorder, characterized by selective loss of motor neurons (MNs). A number of causative genetic mutations underlie the disease, including mutations in the *fused in sarcoma* (*FUS*) gene, which can lead to both juvenile and late-onset ALS. Although ALS results from MN death, there is evidence that dysfunctional glial cells, including oligodendroglia, contribute to neurodegeneration. Here, we used human induced pluripotent stem cells (hiPSCs) with a R521H or a P525L mutation in *FUS* and their isogenic controls to generate oligodendrocyte progenitor cells (OPCs) by inducing *SOX10* expression from a TET-On *SOX10* cassette. Mutant and control iPSCs differentiated efficiently into OPCs. RNA sequencing identified a myelin sheath-related phenotype in mutant OPCs. Lipidomic studies demonstrated defects in myelin-related lipids, with a reduction of glycerophospholipids in mutant OPCs. Interestingly, *FUS*^*R521H*^ OPCs displayed a decrease in the phosphatidylcholine/phosphatidylethanolamine ratio, known to be associated with maintaining membrane integrity. A proximity ligation assay further indicated that mitochondria-associated endoplasmic reticulum membranes (MAM) were diminished in both mutant *FUS* OPCs. Moreover, both mutant *FUS* OPCs displayed increased susceptibility to ER stress when exposed to thapsigargin, and exhibited impaired mitochondrial respiration and reduced Ca^2+^ signaling from ER Ca^2+^ stores. Taken together, these results demonstrate a pathological role of mutant *FUS* in OPCs, causing defects in lipid metabolism associated with MAM disruption manifested by impaired mitochondrial metabolism with increased susceptibility to ER stress and with suppressed physiological Ca^2+^ signaling. As such, further exploration of the role of oligodendrocyte dysfunction in the demise of MNs is crucial and will provide new insights into the complex cellular mechanisms underlying ALS.

**Supplementary Information:**

The online version contains supplementary material available at 10.1007/s00401-023-02666-x.

## Introduction

Amyotrophic lateral sclerosis (ALS) is a progressive neurodegenerative disorder characterized by selective degeneration of upper and lower motor neurons (MNs) in the motor cortex, brainstem, and spinal cord, resulting in fasciculations, spasticity, progressive weakness of muscles, and leading to paralysis and death [19]. ALS is rapidly fatal as the typical time from diagnosis to death is 2–5 years [29]. No effective treatment is available for ALS. Riluzole, an FDA-approved drug which has anti-excitotoxic properties, prolongs life by only a few months [5]. While 90% of ALS cases are sporadic, 10% are inherited. The most common genetic mutations causing ALS are mutations in the ‘*superoxide dismutase 1*’ (*SOD1*), ‘*fused in sarcoma*’ (*FUS*), ‘*TAR DNA binding protein*’ (*TARDBP*) genes, or a hexanucleotide repeat expansion in the ‘*chromosome 9 open reading frame 72*’ (*C9orf72*) gene [92]. ALS-linked *FUS* mutations are highly penetrant and associated with earlier disease onset and more aggressive progression [34, 72]. *FUS* was first identified as an oncogene and was reported as an ALS-causing gene in 2009 [44, 96]. The FUS protein is predominantly nuclear due to its nuclear localization signal (NLS) and can undergo liquid–liquid phase separation due to its prion-like domains [74]. FUS functions as a DNA/RNA-binding protein and is involved in multiple aspects of DNA/RNA metabolism [17]. More than 50 mutations in *FUS* have been identified in families afflicted with ALS [41]. The most common *FUS* mutation affects arginine 521 (R to H, C, or G) [84], and specific missense variants in the NLS (R521H) lead to a milder disease course, with onset in late-adult life, slow progression, and prolonged survival [24]. Another mutation found in the NLS, P525L, causes juvenile-onset ALS which is highly aggressive and penetrant [43], and in some patients, it leads to multiple system degeneration and the presence of basophilic inclusions [15]. The most significant pathological change in postmortem tissue is the cytoplasmic mislocalization of FUS, which is believed to contribute to ALS pathology, possibly via both loss- and gain-of-function mechanism [41]. Although the exact mechanisms responsible for MN degeneration in (FUS-linked) ALS are not known, several functional defects have been described in various models (including among others human or murine spinal cord, murine cell lines, and murine or human stem cell-derived neurons) that may contribute to MN death, including excitotoxicity [14], axonal transport defects [27], mitochondrial abnormalities [70, 100], ER stress [65], and mitochondria-associated ER membrane (MAM) defects [27, 86].

Despite the selective MN degeneration, several studies demonstrated an important role for oligodendrocytes in ALS [36, 48, 71, 80]. Oligodendrocytes, which represent approximately 50–75% of glial cells [4, 95], are myelin-producing cells in the central nervous system (CNS) that originate from precursor cells known as NG2 glia [6]. Oligodendrocyte pathology has been found in multiple areas in postmortem CNS tissue from ALS patients [38, 91], and its relevance is underscored by the consistent presence of FUS inclusions in oligodendrocytes [58]. Most of the evidence for the role of oligodendrocytes in experimental models of ALS was obtained from mouse models. Specifically, oligodendrocyte precursor cells (OPCs) showed increased proliferation and differentiation, and the newly generated oligodendrocytes appeared dysfunctional, resulting in impaired MN insulation and decreased metabolic support [48]. Importantly, selective removal of mutant *SOD1* in oligodendrocytes of *SOD1*^*G93A*^ mice resulted in a delayed disease onset [36]. In another study, conditional depletion of *FUS* in oligodendrocytes led to motor hyperactivity, increased myelin deposition, and cholesterol activation [28].

Although intensive research has been done using different ALS animal models, highly variable phenotypes have been observed. In addition, unless mutations are selectively introduced or removed from different neural cell types, it is not possible to attribute defects in neurons *versus* glial cells to a specific phenotype. The advent of human induced pluripotent stem cell (hiPSC) technology has provided novel tools to recreate human disease models, avoiding different species effects and enabling evaluation of cell-type specific defects generated from patient-derived iPSCs. iPSC-derived MNs, astrocytes, and oligodendroglial progenitors from patients with different causative ALS mutations have been studied and revealed most, if not all, defects thought to be involved in the pathogenesis of ALS [60, 94]. Mutant *FUS* MNs, microglia, and astroglial progenitors have been studied [27, 39, 88], but until now, mutant *FUS* iPSC-derived oligodendroglia have not been evaluated.

In this study, we used iPSCs derived from fibroblasts of an ALS patient carrying the R521H point mutation in the NLS region of FUS, and created an isogenic control line by CRISPR-Cas9. We also introduced the P525L mutation by base editing in a normal donor iPSC line, to create a second pair of isogenic cell lines. To create oligodendroglial progenitor cells (OPCs) we induced expression of the transcription factor SOX10 integrated into the safe harbor locus *AAVS1* [21, 22] of both pairs of mutant *FUS* and isogenic control hiPSCs. We demonstrated that mutant *FUS* OPCs display lipid metabolism defects, increased susceptibility to ER stress inducers, decreased mitochondrial respiration, and diminished physiological Ca^2+^ signaling from ER Ca^2+^ stores, all consistent with MAM disruption. The latter was further proven by a proximity ligation assay (PLA) using antibodies against ER-resident VAMP-associated protein B and C (VABP) and outer mitochondrial membrane-resident phosphatase-interacting protein 51 (PTPIP51).

## Methods

### Cell culture and genome engineering

The following iPSC lines were used in this study: healthy donor SIGi001-A iPSCs (Sigma), patient-derived *FUS*^*R521H*^ hiPSCs, and the genetically corrected isogenic control (referred to as ‘isogenic 1’; used in the study by Guo et al*.* [27]). In addition, the P525L mutation was introduced in the iSOX10 SIGi001-A iPSC (also named ‘Isogenic 2’) via cytosine base editing to create iSOX10-*FUS*^*P525L*^hiPSCs (see Supplementary experimental procedures).

All iPSC lines were cultured at 37 °C and 5% CO_2_ on Matrigel-coated six-well plates (Cornig) with Essential 8™ Flex Medium Kit with 1% penicillin/streptomycin (Gibco) and passaged twice a week using 0.5 mM EDTA (Gibco). All cells were routinely tested for mycoplasma contamination with MycoAlert Mycoplasma Detection Kit (Lonza). Chemicals and reagents used for cell culture were purchased from Thermo Fisher Scientific unless otherwise specified (see Supplementary Table 1).

### Flow cytometry

Cells were detached using Accutase (Gibco) and resuspended in PBS to which the anti-O4-APC antibody (diluted 1/100, Miltenyi) was added. After 15–30 min incubation at 4 °C, cells were washed with PBS, and flow cytometry was performed using the FACSCanto HTS device (BD Biosciences), acquiring 10,000 cells. Data were analyzed using FACSDiva software (version 6.1.2). The list of antibodies can be found in Supplementary Table 1.

### RT-qPCR

RNA was isolated with the Quick-RNA Microprep Kit (Zymo research) according to the manufacturer’s instructions. cDNA was made starting from 500 ng RNA using the SuperScript™ III First-Strand Synthesis System kit (Thermofisher), followed by RT-qPCR using Platinum SYBR Green qPCR Supermix-UDG (Thermofisher). Data are shown as log_2_fold change. Primer sequences can be found in Supplementary Table 2.

### Immunostaining and fluorescence microscopy

Cells were fixed with 4% PFA for 15 min at RT. After washing with PBS, cells were blocked and permeabilized for 20 min at RT with 5% goat serum (Dako) and 0.1% Triton X-100 (Sigma), all diluted in PBS. Next, primary antibodies were diluted in 5% goat serum and added to the cells overnight at 4 °C. The next day, cells were washed with PBS and secondary antibodies diluted in Dako REAL Antibody Diluent (Dako) were added to the cells for 45 min at RT. Afterward, cells were incubated with Hoechst 33342 (Sigma, diluted 1/2000 in Dako REAL Antibody Diluent) for 5 min at room temperature or were applied a drop of ProLong™ Gold Antifade Mountant with DNA Stain DAPI (Thermo Scientific™). The list of antibodies can be found in Supplementary Table 1. Images (MBP^+^ and O4^+^) were acquired using the Operetta High Content Imaging System (PerkinElmer). Consecutive image analysis was performed using automatic and unbiased object segmentation and counting in each channel using the image analysis platform Columbus (Revvity). In short, the nucleus (Hoechst staining) was the first sub-cellular structure to be segmented and used as a reference point for cytoplasm segmentation. Both segmented cell regions were selected for each separate immunofluorescence experiment. Within the selected cell population, the mean fluorescence intensity was used to set a threshold for calculating the percentage of positive cells for each marker.

Images (FUS, VAPB, and PTPIP51 staining) were obtained using a Confocal Nikon C2 microscope and processed with Nikon’s Confocal NIS-Elements imaging software. For the quantification of FUS mislocalization, the customized protocol enables the quantification of protein distributions between cytosol (CellMask) and nucleus (DAPI) using the public domain image processing program ImageJ [26]. For the quantification of VAPB and PTPIP51, the mean intensity was calculated by dividing the fluorescence intensity by the area (in µm2) using ImageJ.

### RNA sequencing

RNA quality control: RNA concentration and purity were determined spectrophotometrically using the Nanodrop ND-1000 (Nanodrop Technologies), and RNA integrity was assessed using a Bioanalyser 2100 (Agilent) and a 5300 Fragment Analyzer (Agilent).

Library preparation: Per sample, an amount of 100 ng of total RNA was used as input. For samples that contained less than 100 ng, all RNA was used. Using the Illumina TruSeq^®^ Stranded mRNA Sample Prep Kit (protocol version: Part 1000000040600v00 October 2017), poly-A-containing mRNA molecules were purified from the total RNA input using poly-T oligo-attached magnetic beads. In a reverse transcription reaction using random primers, RNA was converted into first-strand cDNA and subsequently converted into double-stranded cDNA in a second-strand cDNA synthesis reaction using DNA Polymerase I and RNAse H. The cDNA fragments were extended with a single ‘A’ base to the 3′ ends of the blunt-ended cDNA fragments, after which multiple indexing adapters were ligated, introducing different barcodes for each sample. Finally, enrichment PCR was carried out to enrich those DNA fragments that have adapter molecules on both ends and to amplify the amount of DNA in the library.

Sequencing: Sequence libraries of each sample were equimolarly pooled and sequenced in Illumina HiSeq 4000 (2 lanes, 75 bp, single reads) at the VIB Nucleomics core.

Pre-processing: Low-quality ends and adapter sequences were trimmed off from the Illumina reads with FastX 0.0.14 and Cutadapt 1.15 [59]. Subsequently, small reads (length < 35 bp), poly-A reads (more than 90% of the bases equal A), ambiguous reads (containing N), low-quality reads (more than 50% of the bases < Q25) and artifact reads (all but three bases in the read equal one base type) were filtered using FastX 0.0.14 and ShortRead 1.40.0 [63]. With Bowtie2 2.3.3.1, we identified and removed reads that align to phix_illumina [46].

Mapping: The pre-processed reads were aligned with STAR aligner v2.5.2b to the reference genome of Homo Sapiens (GRCh38) [16]. Default STAR aligner parameter settings were used, except for ‘-outSAMprimaryFlag OneBestScore -twopassMode Basic -alignIntronMin 50 -alignIntronMax 500000 -outSAMtype BAM SortedByCoordinate’. Using Samtools 1.5, reads with a mapping quality smaller than 20 were removed from the alignments [49].

Counting: The number of reads in the alignments that overlap with gene features were counted with featureCounts 1.5.3 [53]. The following parameters were chosen: -Q 0 -s 2 -t exon -g gene_id. Genes for which all samples had less than 1 count-per-million were removed. Raw counts were further corrected within samples for GC content and between samples using full quantile normalization, as implemented in the EDASeq package from Bioconductor [76]. All fastq and supplementary files were uploaded to NCBI-GEO under accession number GSE239403.

Differential gene expression: With the EdgeR 3.24.3 package of Bioconductor, a negative binomial generalized linear model (GLM) was fitted against the normalized counts [78]. We did not use the normalized counts directly, but worked with offsets. Differential expression was tested with a GLM likelihood ratio test, also implemented in the EdgeR package. The resulting *p* values were corrected for multiple testing with Benjamini–Hochberg to control the false discovery rate [31].

Gene set enrichment analysis: To detect enriched functional gene sets, the online tool WebGestalt (http://www.webgestalt.org/) was used with gene sets from the ‘biological process’ gene ontology functional database. Genes were ranked by the signed log of the *p* values from the differential expression analysis. HGNC symbols were used for gene identifiers. The analysis was limited to gene sets with less than or equal to 500 genes and more than or equal to 20 genes.

### Meta-analysis of published RNAseq and CLIPseq data

Data set collection for meta-analysis: Transcriptomic studies publicly available before October 2023 in *Homo sapiens* were identified in the NCBI Sequence Read Archive (SRA; https://www.ncbi.nlm.nih.gov/sra) and Gene Expression Omnibus (GEO; https://www.ncbi.nlm.nih.gov/geo/) databases using the combination of keywords ‘RNA-Seq’, ‘Homo sapiens’, ‘Tissue’, ‘C9orf72’ or ‘Sporadic ALS’. We found two large studies satisfying these criteria. The first study (PRJNA907856) included single-nucleus RNA sequencing (snRNA-seq) of the motor cortex of *C9orf72*-ALS patients and controls (GSE219280). The second study (PRJNA505842 and PRJNA512012) included RNAseq of the motor cortex of sporadic ALS patients and non-neurological controls (GSE122649 and GSE124439). The raw data (GSE122649_RAW.tar, and GSE124439_RAW.tar) were downloaded and subsequently analyzed.

Differential expression analysis for sporadic ALS data was carried out following the procedures outlined in the previous RNA sequencing section. Meanwhile, the differential expression analysis for snRNA-seq data was downloaded from Supplementary Dataset 3 of the corresponding study [50].

Gene set enrichment analyses were performed as described in the previous RNA sequencing section.

The comprehensive list of RNA targets bound by FUS was established by integrating the results of cross-linking and immunoprecipitation (CLIPseq) experiments from three published studies conducted on human samples. This includes HEK293T cells [32], HeLa cells [106], and normal human brain tissue [64]. To analyze FUS splicing targets, we regrouped previously identified and published targets obtained by knock-down or knock-out of FUS in mice and human cells [12, 33, 37, 45, 83]. Notably, the selection criteria for all analyses were set to include only transcripts with an FDR > 0.05. To specifically identify FUS targeted transcripts linked to lipid metabolism, we meticulously curated a list of 429 genes associated with lipid metabolism based on their inclusion in various KEGG pathways (00061; 00062; 00071; 00100; 00120; 00140; 00561; 00564; 00565; 00600; 00590; 00591; 00592; 01040; 04979). Subsequently, we cross-referenced this curated list with the FUS RNA targets and spliced transcripts to facilitate a thorough exploration of potential connections between FUS and lipid metabolism.

### Lipidomics

Lipid extraction: 700 μl of homogenized cells were mixed with 800 μl 1 N HCl:CH_3_OH 1:8 (v/v), 900 μl CHCl_3_ and 200 μg/ml of the antioxidant 2,6-di-tert-butyl-4-methylphenol (BHT; Sigma-Aldrich). 3 μl of SPLASH^®^ LIPIDOMIX^®^ Mass Spec Standard (#330707, Avanti Polar Lipids) was spiked into the extract mix. The organic fraction was evaporated using a Savant Speedvac spd111v (Thermo Fisher Scientific) at RT and the remaining lipid pellet was stored at − 20 °C under argon.

Mass spectrometry: Just before mass spectrometry analysis, lipid pellets were reconstituted in 100% ethanol. Lipid species were analyzed by liquid chromatography electrospray ionization tandem mass spectrometry (LC-ESI/MS/MS) on a Nexera X2 UHPLC system (Shimadzu) coupled with hybrid triple quadrupole/linear ion trap mass spectrometer (6500+ QTRAP system; AB SCIEX). Chromatographic separation was performed on a XBridge amide column (150 mm × 4.6 mm, 3.5 μm; Waters) maintained at 35 °C using mobile phase A [1 mM ammonium acetate in water–acetonitrile 5:95 (v/v)] and mobile phase B [1 mM ammonium acetate in water–acetonitrile 50:50 (v/v)] in the following gradient: (0–6 min: 0% B → 6% B; 6–10 min: 6% B → 25% B; 10–11 min: 25% B → 98% B; 11–13 min: 98% B → 100% B; 13–19 min: 100% B; 19–24 min: 0% B) at a flow rate of 0.7 ml/min which was increased to 1.5 ml/min from 13 min onwards. Sphingomyelin (SM), cholesterol esters (CE), ceramides (Cer), hexose-ceramides (HexCer), and lactose-ceramides (LacCer) were measured in positive ion mode with a precursor scan of 184.1, 369.4, 264.4, 266.4, 264.4, and 264.4, respectively. Triglycerides (TG), diglycerides (DG) and monoglycerides (MG) were measured in positive ion mode with a neutral loss scan for one of the fatty acyl moieties. Phosphatidylcholine (PC), alkylphosphatidylcholine (PC(O-), alkenylphosphatidylcholine (PC(P-), lyso-PC (LPC), phosphatidylethanolamine (PE), alkylphosphatidylethanolamine (PE(O-), alkenylphosphatidylethanolamine (PE(P-), lyso-PE (LPE), phosphatidylglycerol (PG), phosphatidylinositol (PI), and phosphatidylserine (PS) were measured in negative ion mode by fatty acyl fragment ions. Lipid quantification was performed by scheduled multiple reactions monitoring (MRM), the transitions being based on the neutral losses or the typical product ions as described above. The instrument parameters were as follows: curtain gas = 35 psi; collision gas = 8 a.u. (medium); ionspray voltage = 5500 V and − 4500 V; temperature = 550 °C; ion source gas 1 = 50 psi; ion source gas 2 = 60 psi; declustering potential = 60 V and − 80 V; entrance potential = 10 V and − 10 V; collision cell exit potential = 15 V and − 15 V. The following fatty acyl moieties were taken into account for the lipidomic analysis: 14:0, 14:1, 16:0, 16:1, 16:2, 18:0, 18:1, 18:2, 18:3, 20:0, 20:1, 20:2, 20:3, 20:4, 20:5, 22:0, 22:1, 22:2, 22:4, 22:5, and 22:6 except for TGs which considered: 16:0, 16:1, 18:0, 18:1, 18:2, 18:3, 20:3, 20:4, 20:5, 22:2, 22:3, 22:4, 22:5, 22:6.

Data analysis: Peak integration was performed with the MultiQuantTM software version 3.0.3. Lipid species signals were corrected for isotopic contributions (calculated with Python Molmass 2019.1.1). To calculate the concentrations of lipid species per class or lipids with similar acyl chain properties, individual lipid species concentrations from normalized data were summed. Statistical analysis was performed on MetaboAnalyst 5.0 (online software) [68]. First, data were imputed to remove missing values. Lipid species with > 50% missing values were removed, and the remaining missing values were replaced by LoDs (1/5 of the minimum positive value of each lipid species). To perform univariate and multivariate analyses in MetaboAnalyst, data were log_10_ transformed and auto-scaled (mean-centered and divided by the standard deviation of each lipid species). To obtain the overall picture of the data set, all groups were compared by principal component analysis (PCA). Heatmap was used to display the TOP50 most altered lipid species (one-way ANOVA, *p* < 0.05, FDR corrected). Metabolite set enrichment analysis was performed in Metaboanalyst 5.0 based on normalized data (lipids with a fold change (FC) > 2; metabolite set library: chemical structures, main-class).

### Western blot

OPCs were manually collected on ice and fresh-frozen cell samples were stored at − 80 °C until further processing. Cells were washed in DPBS and harvested in a lysis buffer of 10 ml M-PER (Thermo Scientific™), 1 tablet PhosSTOP™ Phosphatase Inhibitor Cocktail (Roche Diagnostics) and 1 tablet complete™ Protease Inhibitor (Roche Diagnostics). Protein concentration was measured using a Pierce™ BCA Protein Assay kit (Thermo Scientific™) as per the manufacturer’s instructions. Samples containing 30 μg protein were supplemented with SDS-containing lane marker reducing sample buffer (Thermo Scientific™), denatured at 95 °C for 10 min and loaded on 4–20% ExpressPlus™ PAGE gels (GenScript). The gel was run at a 120 V for 2 h and then transferred to a 0.2 μm nitrocellulose membrane of a Trans-Blot Turbo Mini Transfer stacks (Invitrogen) using the 7 min, 2.5 A, 25 V program of the iBlot™ 2 dry Blotting Transfer system. The membrane was blocked in 5% skim milk (Sigma-Aldrich) diluted in Tris-buffered saline (Sigma-Aldrich) with Tween (Sigma-Aldrich) (TBST) for 1 h and subsequently incubated overnight with primary antibodies (see Supplementary Table 1) in 5% BSA-TBST at 4 °C. The following day, the membrane was washed three times for 10 min in TBST and incubated with secondary antibodies diluted in TBST for 1 h at RT. Finally, the membrane was washed three times for 10 min in TBST, treated with SuperSignal™ West Pico PLUS Chemiluminescent Substrate (Thermo Scientific™) and imaged with a chemiluminescence ImageQuant LAS4000. Quantification was made with ImageJ.

### X-box-binding protein 1 (XBP1) splicing assay

First, RNA extraction and subsequent cDNA synthesis was performed similarly as described for the RT-qPCR experiments. Next, a PCR reaction was set-up with 140 ng cDNA using the Platinum^®^ Taq mix (Invitrogen™) according to manufacturer’s instructions. Primers used included: human XBP-1, 5′-GGGTTAAGACAGCGCTTGGGGATGG-3′ and 5′-GGGAATCCATGGGGAGATGTTCTG-3′. PCR conditions were: 94 °C for 2 min; 94 °C for 15 s; 59 °C for 30 s; 68 °C for 1 min; 68 °C for 10 min with 30 cycles of amplification. A 289 bp amplicon was generated from unspliced Xbp-1; a 263 bp amplicon was generated from spliced XBP-1. PCR products were resolved on a 2.5% agarose/1 × TAE gel containing SYBR Safe. As previously reported, a minor hybrid amplicon species consisting of unspliced XBP-1 annealed to spliced XBP-1 was also produced through the PCR reaction and appeared above the unspliced amplicon [54]. Quantification of spliced XBP-1 mRNA as a percentage of total XBP-1 (hybrid, unspliced and spliced) mRNA was performed using ImageJ.

### Seahorse analysis

OPCs were plated in a Seahorse XF24 (Agilent) Extracellular Flux Analyzer culture plate in the culture medium overnight. 1 h before the assay, cells were switched to the Mitostress assay medium (Seahorse Biosciences) supplemented with 10 mM glucose, 2 mM glutamine and 1 mM sodium pyruvate, following the manufacturer’s instructions. Oxygen consumption rate (OCR) during the Mitostress assay was measured with the Seahorse XF24 (Agilent) Extracellular Flux Analyzer (Seahorse Biosciences) following the manufacturer’s instructions. Following the Mitostress assay, 1 μM oligomycin, 2.0 μM FCCP (Isogenic 2 and *FUS*^*P525L*^) or 2.5 μΜ FCCP (Isogenic 1 and *FUS*^*R521H*^) and 0.5 μM antimycin A diluted in assay medium were the final concentration in the wells. Once the run was finished, all the medium was removed from the well and all the cells were collected and counted by ChemoMetec cell counter 900–002 NucleoCounter.

### Ca^2+^ release studies

OPCs were plated in a four-chamber glass bottom dish (Cellvis) 4 days prior to the measurements. On the day of the recordings, cells were loaded with 1.25 μM Cal-520AM in medium in a humidified incubator (37 °C with 5% CO2). After 30 min, the cells were washed twice with PBS+/+ solution followed by a 30 min incubation/de-esterification step in the incubator in medium. Following de-esterification, the medium was changed to a modified Krebs–Ringer solution (135 mM NaCl, 6.2 mM KCl, 1.2 mM MgCl_2_, 12 mM HEPES, pH 7.3, 11.5 mM glucose and 2 mM CaCl_2_) in which all subsequent stimuli were also solubilized. After establishing a 30 s baseline each measurement, the different stimuli were added and responses were measured. For quantifying ATP (10 μM) and acetylcholine (Ach) (10 μM) responses, both AUC and peak of the responses were determined. Changes in Cal-520 fluorescence intensity were measured at 510 nm with excitation at 480 nm using a Nikon eclipse Ti2 inverted fluorescence microscope (Nikon) equipped with excitation filter FF01-378/474/554/635 and dichroic mirror FF01-432/515/595/730 and emission filter 515/30, all from Semrock. Excitation was performed at 470 nm using a CoolLed pR-4000 (CoolLed). Acquisition of the emitted fluorescent signal was performed at 10 Hz using a pco.edge 4.2bi sCMOS camera (pCO). Fluorescence signals were plotted as *F*/*F*_0_, where *F*_0_ was the averaged signal from the first ten baseline images.

### Proximity ligation assay

PLA was performed using the Duolink In Situ Detection Reagent Red, Sigma-Aldrich, DUO92008; Duolink In Situ PLA Probe Anti-Mouse MINUS, Sigma-Aldrich, DUO92004; Duolink In Situ PLA Probe Anti-Rabbit PLUS, Sigma-Aldrich, DUO92004; Duolink In Situ Mounting Medium with DAPI, Sigma-Aldrich, DUO82040; according to the manufacturer’s instructions. Briefly, OPCs were seeded into a CultureWell™ Chambered Coverglass (16 wells per coverglass), fixed using 4% PFA for 10 min and permeabilization with 0.1% Triton. Wells were then incubated with the primary antibodies, anti-protein tyrosine phosphatase-interacting protein 51 (PTPIP51) (rabbit; ab224081), and anti-vesicle-associated membrane protein B (VAPB) (mouse; MAB58551), in blocking solution (PBS + 0.1% Triton X-100 + 4% BSA). Secondary antibodies PLUS and MINUS (anti-rabbit and anti-mouse IgG antibodies conjugated with oligonucleotides) were incubated 1:5 in blocking solution for 1 h at 37 °C. The ligation solution (ligation buffer 1:5, ligase 1:40 in MQ) was then applied for 30 min at 37 °C. The amplification solution (amplification buffer 1:5, polymerase 1:40 in MQ) was applied for 1 h 40 min at 37 °C. After incubation, slides were mounted with Duolink In Situ Mounting Medium with DAPI. As a negative control, we incubated VAPB alone with both PLUS and MINUS secondary antibodies. Images were obtained using a Confocal Nikon C2 microscope and processed with Nikon’s Confocal NIS-Elements imaging software. The customized protocol enabled the quantification of interactions detected by PLA using the public domain image processing program ImageJ [56].

### Statistical analysis

All statistical analyses were carried out using Graphpad Prism version 8.3.0. Graph creation was performed using Inkscape 1.3. Each experiment conducted in this study was repeated at least three times. The variable ‘*N*’ denotes the number of independent differentiations, where indicated that experiments represent, e.g., *N* = 3; this signifies that studies were done using OPCs generated from a different vial of iPSC from the given cell line. For the analysis of immunostaining and proximity ligation assay, ‘*N*’ denotes the number of images per condition. To compare differences between mutant *FUS* and isogenic control groups, unpaired two-tailed *t* test were used, while one-way ANOVA or two-way ANOVA with a Bonferroni's multiple comparisons was used to assess difference between multiple groups. Significance levels are indicated with asterisks (*p < 0.05, **p < 0.01, ***p < 0.001, ****p < 0.0001). All values are given as mean ± standard deviation (SD).

## Results

### Genome engineering to create ***FUS***^***R521H***^ and ***FUS***^***P525L***^ iPSCs as well as isogenic controls containing an inducible ***SOX10*** transcription factor cassette

To create OPCs efficiently by SOX10 overexpression, we introduced a *TET-ON-SOX10* cassette in the *AAVS1* locus of both the *FUS*^*R521H*^ iPSC (*FUS*^*R521H*^) and its isogenic control line (Isogenic 1) as well as the SIGi001-A iPSCs (Isogenic 2), by sequential introduction of an FRT-site flanked cassette followed by recombination-mediated cassette exchange of the FRT-flanked *TET-ON-SOX10* cassette as described before [22, 66] (Supplementary Fig. 1a). As quality control, we performed Southern blot analysis to demonstrate absence of random integrants, 5’ junction PCR to demonstrate insertion of the cassette in the *AAVS1* locus, and inducibility of the iSOX10 cassette by RT-qPCR (Supplementary Fig. 1b–e).

To create the *FUS*^*P525L*^* iSOX10*-iPSC, the P525L *FUS* mutation was introduced in iSOX10 SIGi001-A iPSCs (Isogenic 2) using cytidine deaminase base editing using AncBE4max which is an improved cytidine base editor due to higher expression, improved NLS, codon usage, and entails ancestral reconstruction of the deaminase component. The base editing workflow was performed following the method described in Nami et al., 2021 (Supplementary Fig. 2a). Briefly after nucleofection of the iPSCs with a sgRNA targeting the endogenous *FUS* locus and the AncBE4max-P2A-GFP plasmid, GFP^+^ cells were sorted to select for successfully transfected cells [42]. Afterward, iPSC colonies were isolated and screened using Sanger sequencing to identify clones wherein the *FUS*^*P525L*^ mutation had been correctly introduced (Supplementary Fig. 2b). Sanger sequencing of in silico predicted off-target regions demonstrated no off-target events (Supplementary Fig. 2c).

### Presence of *FUS* mutations does not affect OPC differentiation

**Fig. 1:**
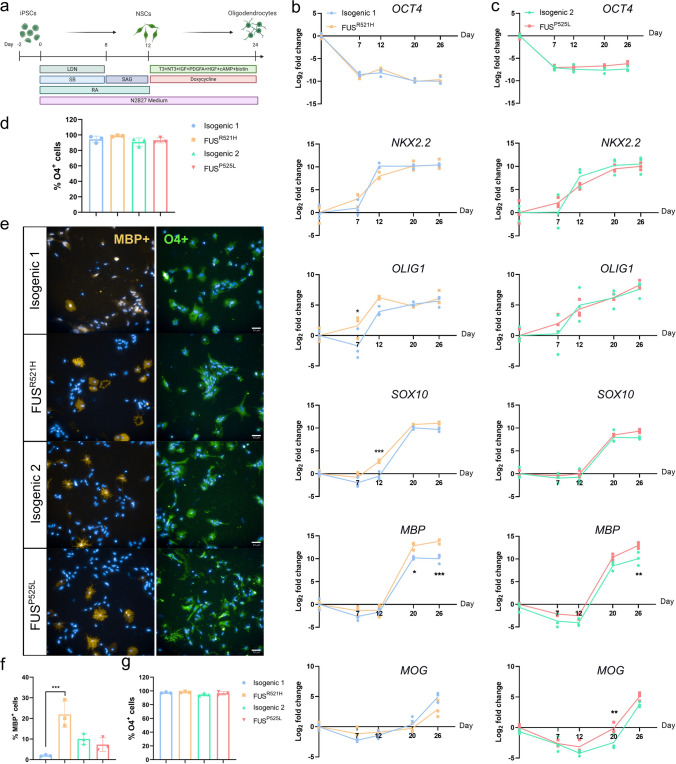
Presence of mutant *FUS* does not affect iPSC-derived OPC differentiation. **a** Schematic overview of the oligodendrocyte differentiation protocol. RT-qPCR of *OCT4*, *NKX2.2*, *OLIG1*, *SOX10*, *MBP* and *MOG* transcripts throughout the oligodendrocyte differentiation of *FUS*^*R521H*^ mutant and isogenic control (**b**) and *FUS*^*P525L*^ mutant and isogenic control (**c**) (*N* = 3 independent differentiations). **d** Determination by flow cytometry of the percentage of O4^+^ cells in DIV26 OPCs generated by SOX10 overexpression from the *AAVS1* locus (*N* = 3 independent differentiations). **e** Immunofluorescent staining for MBP and O4 on DIV26-30 OPCs generated from *FUS*^*P525L*^, *FUS*^*R521H*^ mutant and the isogenic control hiPSCs (scale bar: 50 μm). Quantification of the percentage of MBP (**f**) and O4 (**g**) positive cells. Data are represented as mean ± SD of three biological replicates (*n* = 33 images). Statistical analyses were performed by one-way ANOVA (**d**–**g**) and two-way ANOVA (**b**, and **c**) with the Bonferroni's multiple comparisons test. Data are represented as mean ± SD. *p < 0.05 **p < 0.01 ***p < 0.001

Both pairs of mutant and isogenic non-mutant hiPSCs were differentiated toward OPCs as described in Garcia-Leon et al*.* [21, 22] (Fig. 1a). Transcript levels of the pluripotency gene *OCT4* decreased in all hiPSC lines, while oligodendrocyte-related transcripts (*NKX2.2*, *OLIG1*, *SOX10*, *MBP*, and *MOG*) progressively increased (Fig. 1b, c). Remarkably, mutant *FUS* OPCs exhibited markedly elevated transcript levels of *MBP* and *MOG* in comparison to their isogenic non-mutant counterparts (*p* < 0.05) (Fig. 1b, c). Flow cytometry demonstrated that all OPCs at day 24 were nearly 100% O4 positive (Fig. 1d), which was consistent with the immunofluorescence imaging for O4 (Fig. 1e, g). Immunostaining for MBP revealed that significantly more *FUS*^*R521H*^ OPCs stained positive compared to isogenic corrected OPCs (*p* < 0.01), but this difference was not observed for OPCs carrying the *FUS*^*P525L*^ mutation (Fig. 1f).

### Cytoplasmic FUS accumulation in mutant *FUS* OPCs

**Fig. 2:**
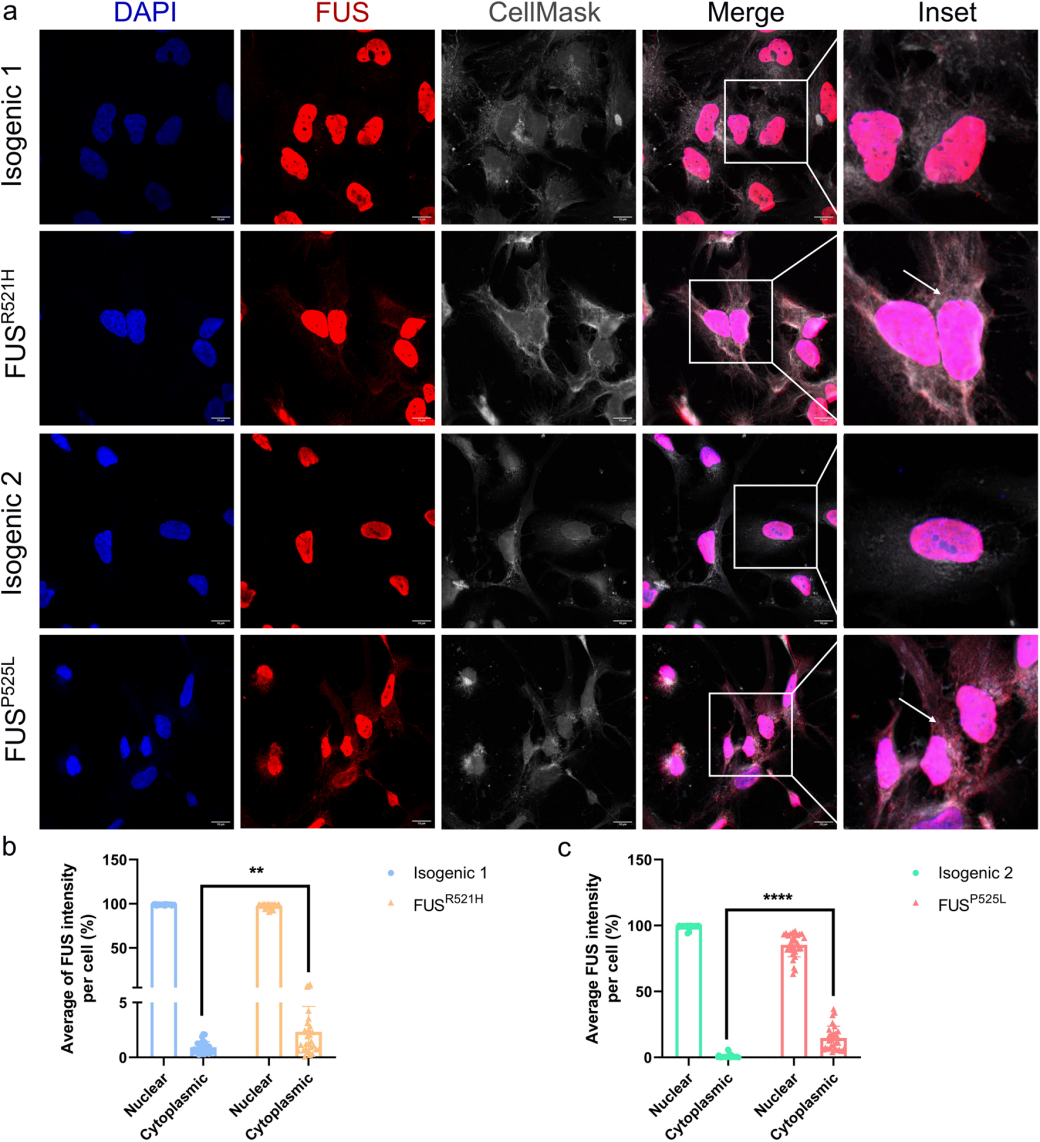
Cytoplasmic FUS accumulation in mutant *FUS* iPSC-derived OPCs. **a** Representative confocal images of FUS protein cellular localization on DIV26-30 OPCs generated from *FUS*^*P525L*^, *FUS*^*R521H*^ and their isogenic control hiPSCs (scale bar: 10 μm). Nuclei are stained with DAPI and cytoplasm with CellMask. Arrows in the inset indicate FUS mislocalization in the cytoplasm of mutant *FUS* OPCs. Quantification of nuclear and cytoplasmic *FUS* staining in *FUS*^*R521H*^ mutant OPCs (**b**) and *FUS*^*P525L*^ mutant OPCs (**c**) and their respective isogenic controls. Statistical analyses were performed by unpaired two-tailed *t* tests. Data are represented as mean ± SD from three biological replicates with three technical replicates in each experiment (*n* = 30 images and n > 150 nuclei/condition). **p < 0.01 ****p < 0.0001

FUS, which is a nuclear protein, is known to be mislocalized to the cytoplasm in ALS neurons and glial cells [27, 88]. We stained the OPCs using an anti-FUS antibody and CellMask™ Plasma Membrane Stains. Mutant *FUS* OPCs exhibited more cytoplasmic FUS aggregates compared to their isogenic controls (Fig. 2a). We calculated the percentage of cells observed with nuclear or cytoplasmic FUS staining in OPCs, which is the fluorescence intensity of the FUS protein in the nucleus or cytoplasm divided by the total intensity of the cell (Fig. 2b, c). This confirmed a significantly increased presence of FUS protein in the cytoplasm of mutant *FUS* OPCs compared to their isogenic controls.

The homeostasis of FUS protein levels is controlled by autoregulatory mechanisms involving the retention of intron 6 and 7 [33, 81]. Furthermore, mutations in the NLS domain lead to the reduction of FUS exon 6 and 6 repression, contributing to FUS abnormal cytoplasmic accumulation [33, 81]. Using RT-PCR, we calculated the percentage of intron 6/7 retention in mutant *FUS* OPCs and their isogenic controls. As shown in Supplementary Fig. 3a and quantified in Supplementary Fig. 3b, c, a significantly decreased percentage of intron 6 and intron 7 retention was identified in mutant *FUS* OPCs. These results indicated altered FUS autoregulatory mechanisms due to mutations in the NLS domain of the human FUS gene. Increasing *FUS* mRNA production could contribute to cytoplasmic FUS accumulation observed by immunocytochemistry (Fig. 2).

### Transcriptome analysis revealed a myelin sheath signature in mutant *FUS* OPCs

**Fig. 3:**
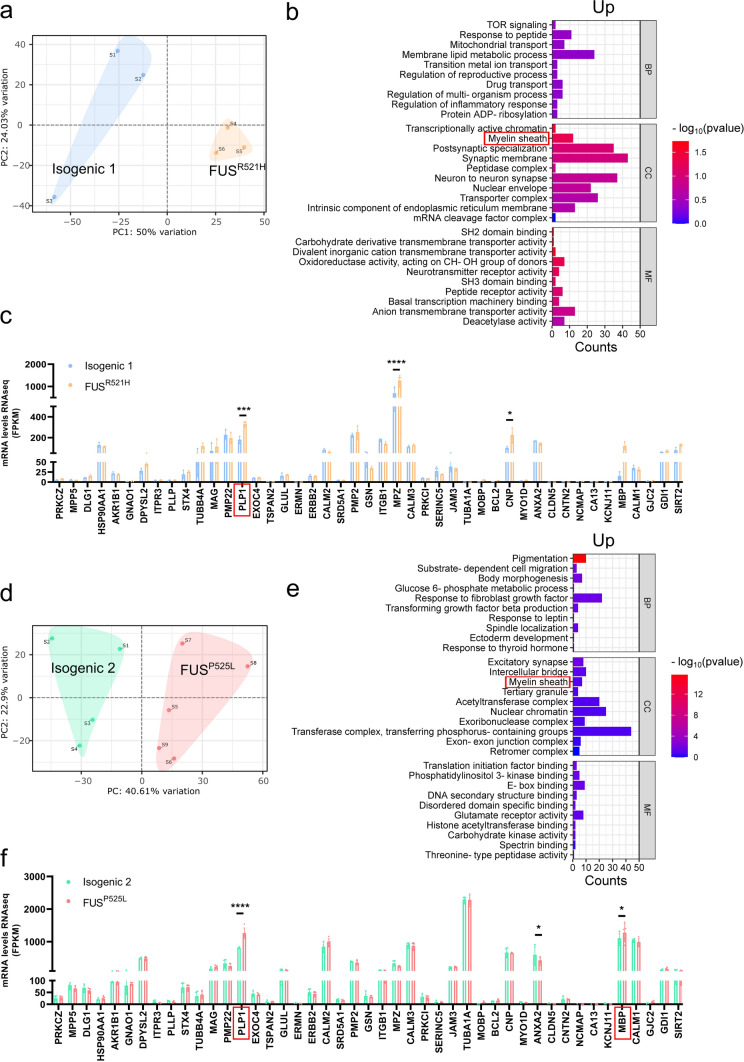
Transcriptome analysis identified myelin sheath pathological signature in mutant *FUS* iPSC-derived OPCs. Principal component analysis (PCA) of *FUS*^*R521H*^ mutant (**a**) and *FUS*^*P525L*^ mutant (**d**) and their respective isogenic controls (*N* ≥ 3 independent differentiations). Ranked gene set enrichment analysis (GSEA) was performed in *FUS*^*R521H*^ mutant OPCs (**b**) and *FUS*^*P525L*^ mutant OPCs (**e**) and their respective isogenic controls using the online tool WebGestalt. The top ten significantly enriched gene ontology (GO) terms of upregulated genes in biological process (BP), cellular component (CC), and molecular function (MF) are presented. Normalized expression from RNAseq data of myelin sheath-related genes in *FUS*^*R521H*^ mutant (**c**) and *FUS*^*P525L*^ mutant (**f**) (N ≥ 3 independent differentiations). Statistical analyses were performed by two-way ANOVA with the Bonferroni’s multiple comparisons test (**c** and **f**). Data are represented as mean ± SD. *p < 0.05; **p < 0.01 ****p < 0.0001

PCA of RNA sequencing data of mutant and isogenic non-mutant OPCs, demonstrated that *FUS*^*R521H*^ and *FUS*^*P525L*^ OPCs clustered separately from their isogenic controls (Fig. 3a, d). In the gene set enrichment analysis (GSEA) with gene ontology (GO), conducted on all genes ranked from most significantly upregulated to most significantly downregulated, among the upregulated genes, one of the identified enriched cellular components was the ‘myelin sheath’ (Fig. 3b, e). Among the downregulated genes, the analysis revealed the top ten significantly differentially expressed pathways associated with GO terms (Supplementary Fig. 4). The GO term ‘myelin sheath’ comprises genes encoding crucial proteins for the formation and maintenance of the lipid-rich myelin sheath, including, among others, *MBP* and *PLP1*, which were more highly expressed in mutant *FUS* OPCs (Fig. 3c, f).

To further determine the relevance of this myelin sheath-related transcriptomic signature, we analyzed publicly available RNA sequencing studies from ALS patients. Because of the lack of data for *FUS*-ALS patients, we focused our analysis on a group of patients with *C9orf72* mutations [50], and a group of sporadic ALS patients [91]. We evaluated the snRNA-seq datasets (GSE219280) of neurons and glia, focusing on the OPC/oligodendrocyte cell cluster present in the motor cortex of six patients with *C9orf72*-ALS, compared with five controls. We identified 166 and 423 genes upregulated in *C9orf72*-ALS OPCs and oligodendrocytes, respectively, with 225 and 201 genes downregulated compared to their respective controls (Supplementary Fig. 5a, d) (FC > 1.2, FDR < 0.05). GSEA illustrated the top ten most dysregulated pathways (Supplementary Fig. 5b, c, e, f). However, we observed that genes involved in the myelin sheath were downregulated in OPCs/oligodendrocytes from *C9orf72*-ALS patients when compared to their respective controls (Supplementary Fig. 5 g, h). GSEA analysis of the RNAseq studies (GSE122649 and GSE124439) performed on the motor cortex of sporadic ALS patients, compared with non-neurological controls, revealed enrichment in several categories, including the cellular component ‘myelin sheath’ and the biological process ‘ensheathment of neurons’ (Supplementary Fig. 6b). Furthermore, we observed higher expression of *MBP* and *PLP1* in sporadic ALS patient samples compared to controls (Supplementary Fig. 6d).

In a similar manner, we analyzed the RNAseq data described by Rossaert et al*.* [79], of *FUS*^+*/*+^ mice wherein a wild-type human FUS has been introduced under the prion promotor. Such animals develop a complete paralysis and premature death around 60 days of age [5]. We identified 714 genes upregulated in the spinal cord of *FUS*^+*/*+^ mice, with 193 genes downregulated compared to wild type (Supplementary Fig. 7a) (FC > 2, FDR < 0.1). GSEA was performed on all genes, ranked from most significantly upregulated to most significantly downregulated, and we employed bar plots to illustrate the top ten most dysregulated pathways (Supplementary Fig. 7b, c). Interestingly, this analysis highlighted the myelin sheath as a top downregulated hit for cellular component. However, in contrast to our results in mutant *FUS* OPCs, we observed decreased expression of *Mbp* and *Plp1* in *FUS*^+*/*+^ mice compared to wild type (Supplementary Fig. 7d).

### Lipid metabolism defects in mutant *FUS* OPCs

**Fig. 4:**
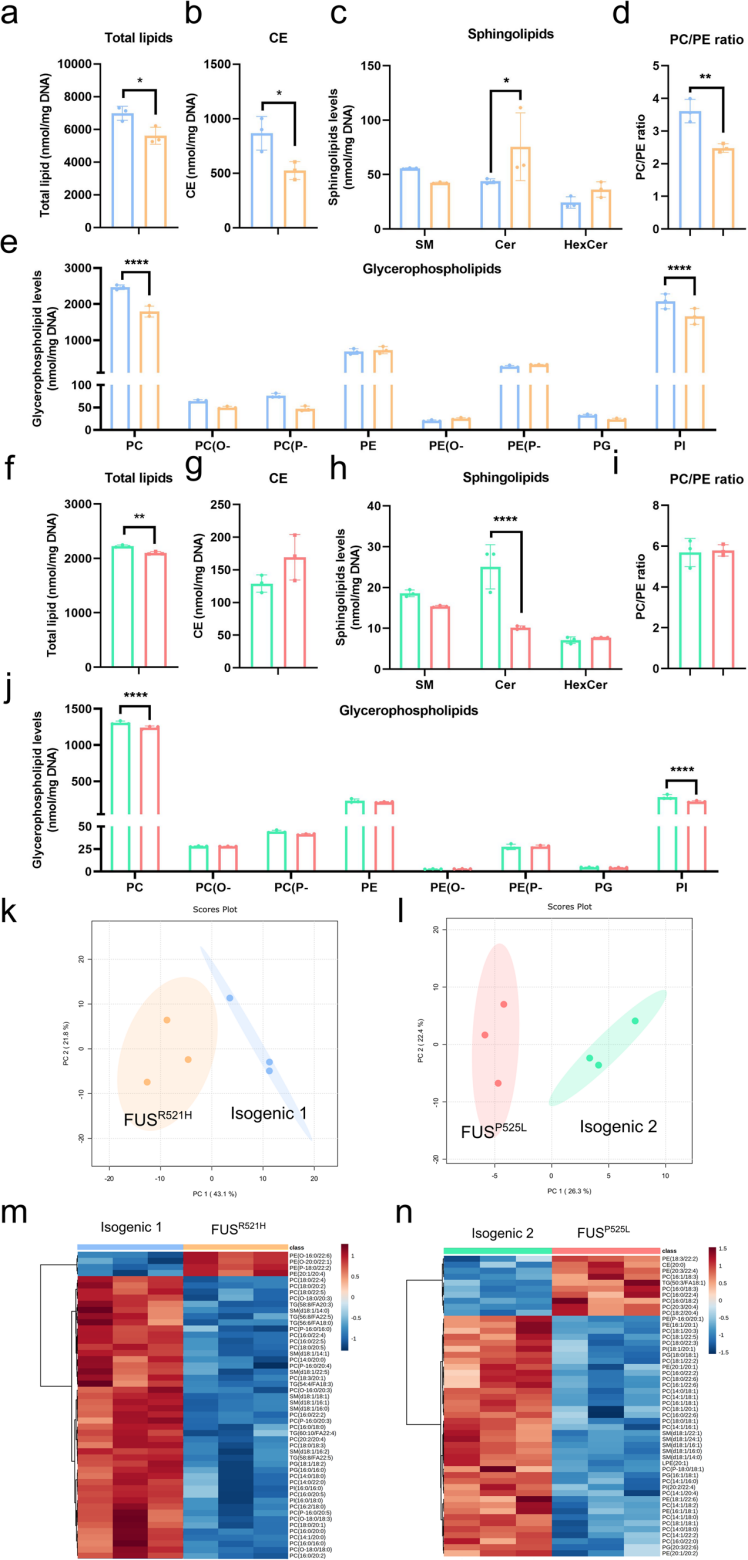
Lipidome analysis identified lipid metabolism defects in *mutant FUS* iPSC-derived OPCs. Quantification of total lipid concentrations (nmol/mg DNA) by mass spectrometry in *FUS*^*R521H*^ mutant OPCs (**a**) and *FUS*^*P525L*^ mutant OPCs (**f**) and their respective isogenic controls (*N* = 3 independent differentiations). Quantification of cholesterol ester (CE) and sphingolipid concentrations (SM, Cer, and HexCer) in *FUS*^*R521H*^ mutant OPCs (**b**, **c**) and *FUS*^*P525L*^ mutant OPCs (**g**, **h**) and their respective isogenic controls. Quantification of glycerophospholipid subclasses (PC, PC(O-), PC(P-), PE, PE(O-), PE(P-), PG, and PI) in *FUS*^*R521H*^ mutant OPCs (**e**) and *FUS*^*P525L*^ mutant OPCs (**j**) and their respective isogenic controls. PC to PE concentration ratio in *FUS*^*R521H*^ mutant OPCs (**d**) and *FUS*^*P525L*^ mutant OPCs (**i**) and their respective isogenic controls. PCA plot of lipidomic data in *FUS*^*R521H*^ mutant OPCs (**k**) and *FUS*^*P525L*^ mutant OPCs (**l**) and their respective isogenic controls. **m**, **n** Heatmap representing all clustered samples and color-coded normalized concentrations of the top 50 most significantly altered individual lipid species (one-way ANOVA, *p* < 0.05, FDR corrected). (PC, phosphatidylcholine; PC(O-), alkylphosphatidylcholine; PC(P-), alkenylphosphatidylcholine; PE, phosphatidylethanolamine; PE(O-), alkylphosphatidylethanolamine; PE(P-), alkenylphosphatidylethanolamine; PG, phosphatidylglycerol; PI, phosphatidylinositol; SM, sphingomyelin; Cer, ceramide; HexCer, hexose-ceramides). Statistical analyses were performed by unpaired two-tailed *t* tests to compare *FUS*-mutant OPCs and their respective controls (**a**, **b**,** d**,** f**,** g**, and** i**) and two-way ANOVA with the Bonferroni’s multiple comparisons test (**c**,** e**,** h**, and** j**). Data are represented as mean ± SD. *p < 0.05 **p < 0.01 ****p < 0.0001

The myelin sheath is mostly made of lipids, including phosphatidylcholine (PC), and phosphatidylethanolamine (PE), critical for its structure and function. To determine if the *FUS* mutation leads to aberrant lipid composition of OPCs, we performed mass spectrometry–based lipidomic analyses of mutant *FUS* and isogenic control OPCs. Higher total lipid levels (normalized for DNA content) were observed in the *FUS*^*R521H*^ pair compared to the *FUS*^*P525L*^ pair, which may be attributed to the relatively larger size of OPCs carrying the *FUS*^*R521H*^ OPCs (Fig. 2a). The overall lipid level in mutant *FUS* OPCs was significantly lower than that of isogenic non-mutant controls (Fig. 4a, f). The cholesterol ester (CE) levels in *FUS*^*R521H*^ mutant OPCs were significantly lower than in isogenic non-mutant controls (Fig. 4b), which was not seen in the *FUS*^*P525L*^ pair (Fig. 4g). However, we observed a significant decrease in the level of PC and phosphatidylinositol (PI) in both mutant *FUS* OPCs (Fig. 4e, j), compared to isogenic non-mutant cells. Interestingly, there was an inverse trend observed in the levels of sphingolipid subclass ceramide (Cer) in both OPC pairs (Fig. 4c, h).

A balanced ratio between the relative abundance of PC and PE is crucial for the maintenance of cell homeostasis, ER and mitochondrial functions, and energy metabolism [99]. We observed a significant decrease in the PC/PE ratio in *FUS*^*R521H*^ OPCs compared to isogenic control (Fig. 4d), but not in *FUS*^*P525L*^ OPCs (Fig. 4i).

To visualize the variation and highlight the robust phenotypes, we conducted a multivariate analysis using Metaboanalyst 5.0 based on normalized data. PCA displayed on two-dimensional score plots (Fig. 4k, i), and heatmap plots (Fig. 4m, n), showed the segregation of 4 groups among the 12 samples analyzed. Samples of the same genotype clustered together (Fig. 4k, i). Among the top 50 most significantly altered individual lipid species, PC species accounted for 21/50 in *FUS*^*R521H*^ versus isogenic OPCs (Fig. 4m), and 22/50 in *FUS*^*P525L*^* versus* isogenic OPCs (Fig. 4n), most of which decreased whereas several PE species increased. This is consistent with the overall decrease in PC levels and unaltered or increased PE in Fig. 4.

### Modifications of glycerophospholipid metabolism in mutant *FUS* OPCs

**Fig. 5:**
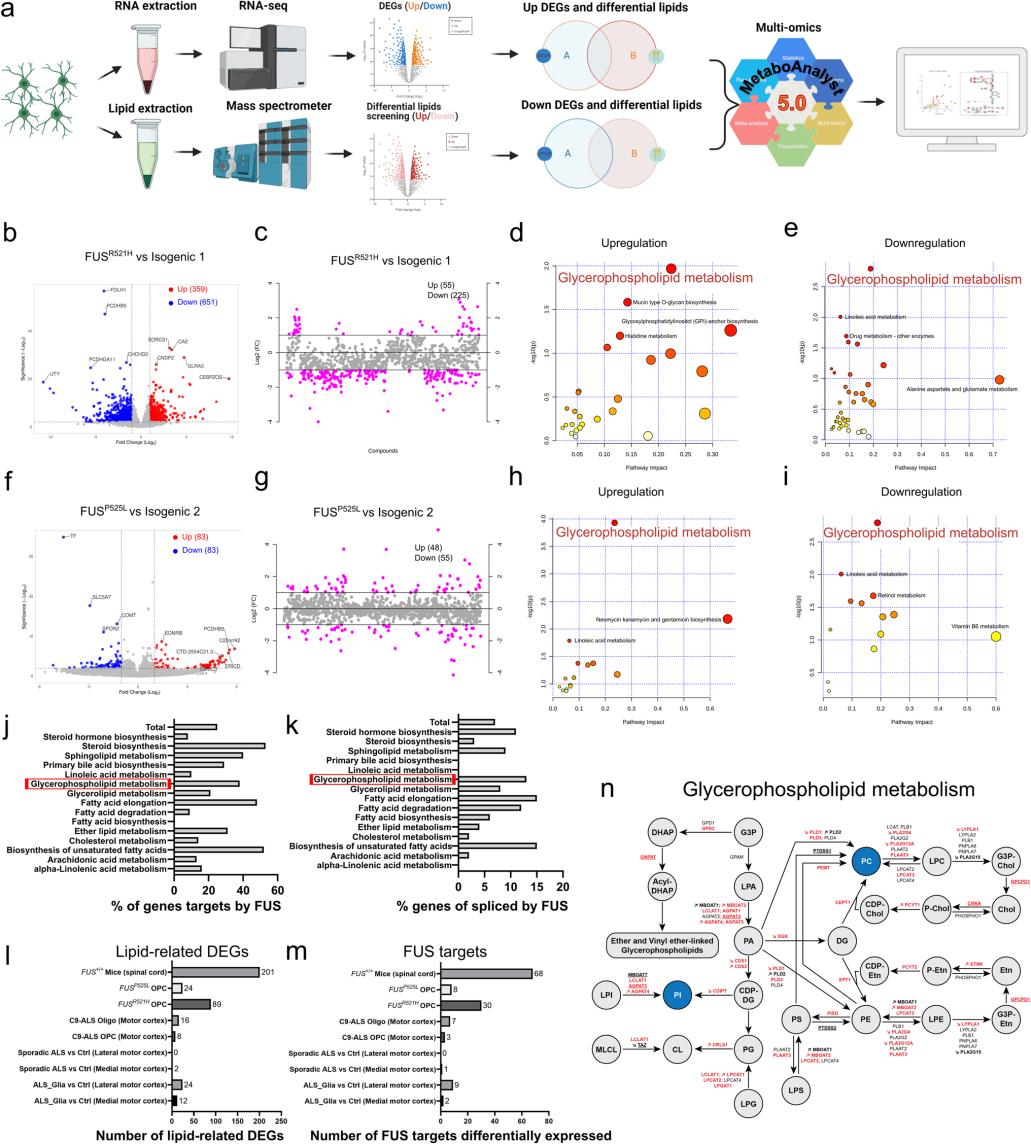
Dysregulated glycerophospholipid metabolism in mutant *FUS* iPSC-derived OPCs. **a** Scheme of joint-pathway analysis. **b**, **f** Volcano plots of upregulated (red) and downregulated (blue) genes in *FUS*-mutant OPCs, compared to isogenic controls. Genes with log_2_FC < − 1.0 and − log_10_(*p*) > 2.0 were considered downregulated and log_2_FC > 1.0 with − log_10_(*p*) > 2.0 were considered upregulated. **c**,** g** Important dysregulated lipid species (red circles) selected by fold-change analysis. Both upregulated (log_2_FC > 1.0) and downregulated (log_2_FC < − 1.0) features are plotted in a symmetrical way. **d**, **h**,** e**,** i** Joint-pathway analysis (MetaboAnalyst v.5.0) shows upregulated and downregulated metabolic pathways in *FUS*-mutant OPCs, compared to isogenic controls. Each circle signifies a distinct pathway, with its size and shade reflecting the pathway’s impact and statistical significance (red denotes the highest significance). **j**, **k** Bar graphs displaying the percentage of known genes targeted by FUS (**j**) or spliced by FUS (**k**) within the different lipid-metabolism-related KEGG pathways (based on studies [12, 33, 37, 45, 64, 83]. **l**, **m** Bar graphs indicating the number of lipid-metabolism-related dysregulated genes (DEGs) (**l**) and the number of lipid-metabolism-related dysregulated genes that can be regulated by FUS (**m**) across previously published datasets compared to *FUS*-mutant OPCs. These datasets include bulk RNAseq data of the spinal cord from symptomatic *FUS*^+*/*+^ mice overexpressing wild-type human FUS compared to wild-type mice [79], single nuclei RNAseq data of primary human motor cortex OPCs/oligodendroglia of *C9orf72*-ALS patients compared with control human brain [50] and bulk RNAseq data of sporadic ALS patient motor cortex compared to control non-ALS control individuals [91], as well as between the so-called motor cortex of a ‘ALS_glia’ subtype group (identified by enriched astroglia, microglia and oligodendroglia dysregulated genes, and this even if—according to the authors—there was no selective neuronal loss) versus motor cortex all other sporadic ALS patients. **n** Scheme of glycerophospholipid metabolism, in which circles indicate metabolites and arrows indicate the enzymatic reaction with the gene name encoding the enzyme. Altered lipid classes in *FUS*-mutant OPCs are highlighted in blue, and arrows indicate whether the gene is upregulated or downregulated based on RNAseq data. Genes targeted by FUS are highlighted in red, while genes spliced by FUS are underlined

To gain mechanistic insights into the dysregulated lipids and genes, we performed joint-pathway analysis using Metaboanalyst 5.0 based on normalized data (Fig. 5a). Overall, 359 and 83 genes were shown upregulated in *FUS*^*R521H*^ and *FUS*^*P525L*^ OPCs, respectively, while 651 (R521H) and 83 (P525L) genes were downregulated in comparison to their respective controls (Fig. 5b, f). Next, we used volcano plots to display the most dysregulated lipids among the whole lipidome, with an adjusted *p* < 0.05 and a FC > 2.0 (Fig. 5c, g). There were 55 and 48 lipids significantly increased in *FUS*^*R521H*^ and *FUS*^*P525L*^ OPCs, respectively, while 225 (R521H) and 55 (P525L) lipids were decreased in comparison to their respective controls (Fig. 5c, g). Next, all differential genes and lipids exhibiting similar trends in each pair were uploaded and analyzed separately to integrate metabolomics data with transcriptomics data [68]. Interestingly, glycerophospholipid metabolism was identified in both pairs of OPCs (Fig. 5d, e, h, i).

To address a potential link between FUS functions and aberrant lipid metabolism, we investigated the nature of previously identified RNA transcripts bound or spliced by FUS. We analyzed human FUS cross-linking and immunoprecipitation (CLIP) data from three different studies [32, 64, 106], and we investigated the splicing targets of FUS*,* based on five different studies [12, 33, 37, 45, 83]. We found that FUS binds the RNA of 7350 genes, among which 6812 code for proteins, and that 1403 genes are spliced by FUS, among which 1370 code for proteins. Strikingly, 25% of genes involved in lipid metabolism are direct FUS targets (Fig. 5j) and 7% are spliced by FUS (Fig. 5k), based on KEGG pathways. Furthermore, we found that genes targeted or spliced by FUS are involved in almost all steps of the glycerophospholipid metabolism (Fig. 5n), indicating an important role of FUS in this metabolic pathway.

Furthermore, we found 89 and 24 lipid-related genes aberrantly expressed in *FUS*^*R521H*^ and *FUS*^*P525L*^ OPCs, respectively (Fig. 5l). Importantly, 30 lipid metabolism genes that can be regulated by FUS were found aberrantly expressed in the mutant *FUS*^*R521H*^ OPCs and 8 in the mutant *FUS*^*P525L*^ OPCs (Fig. 5m). We compared the number of lipid-related DEGs in the previously analyzed data sets and found that the spinal cord of symptomatic *FUS*^+*/*+^ mice contains the highest number of lipid-related DEGs, with a total of 201 (Fig. 5l). Remarkably, we identified 68 lipid metabolism genes that can be regulated by FUS differentially expressed compared to their wild type counterpart (Fig. 5m). When we analyzed the snRNAseq data of OPCs/oligodendroglia from the motor cortex of patients with *C9orf72* mutant ALS, among 8 and 16 lipid-related DEGs (Fig. 5l), 3 and 7 lipid metabolism genes that can be regulated by FUS, respectively, were found deregulated (Fig. 5m). The analysis of sporadic ALS patient’s data did not reveal dysregulated genes linked to lipid metabolism. However, when we analyzed the so-called ‘ALS_glia’ subtype group (where RNAseq identified mainly deregulated astroglia, microglia and oligodendroglia genes to be deregulated, and this even if according to the authors there was no selective neuronal loss), compared with controls, among 12 and 24 lipid-related DEGs (Fig. 5l), 2 and 9 genes that can be regulated by FUS were found differentially expressed in the medial and lateral cortex, respectively (Fig. 5m).

### MAM disruption in mutant *FUS* OPCs

**Fig. 6:**
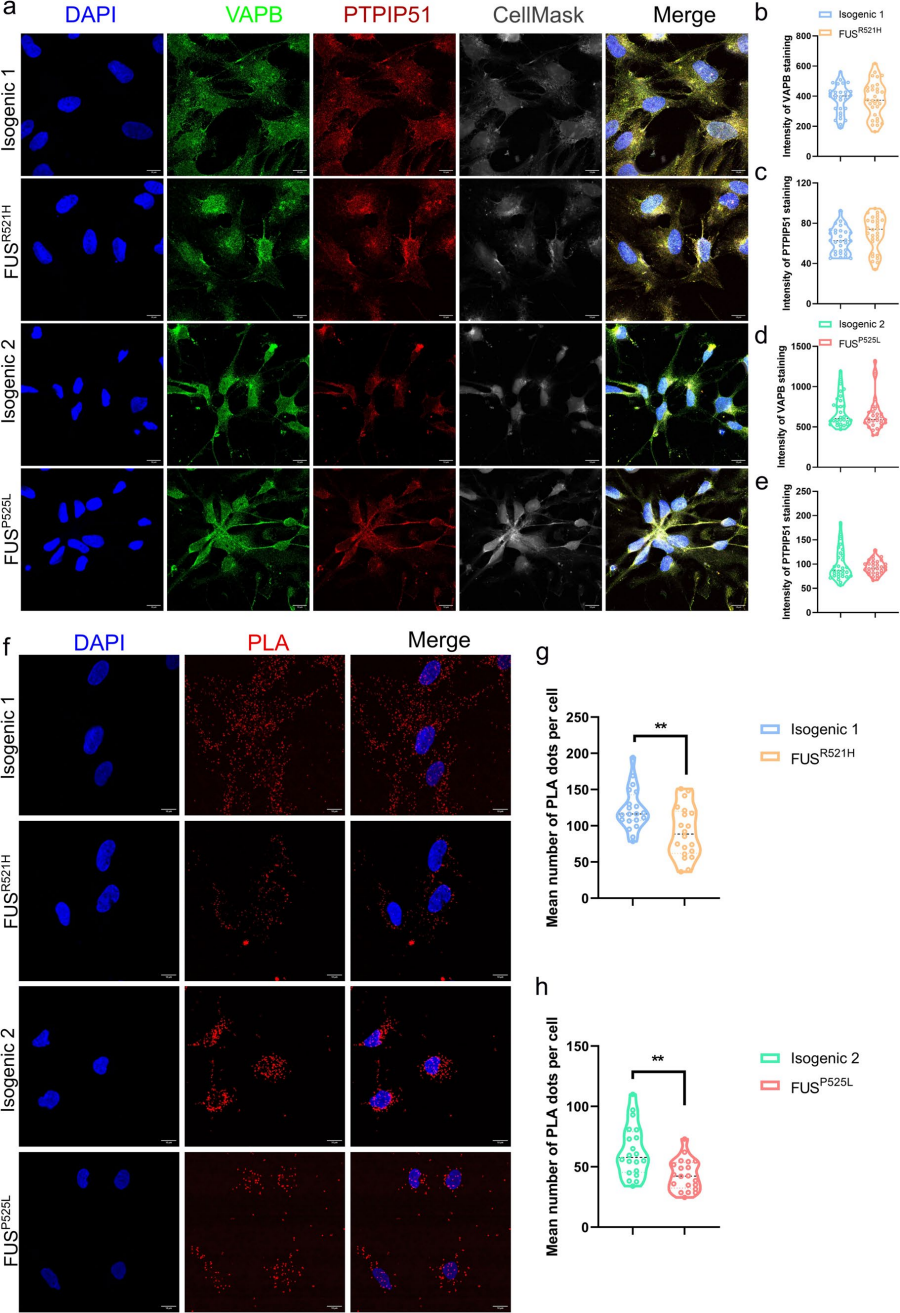
MAM disruption in mutant *FUS* iPSC-derived OPCs. **a** Immunofluorescent staining for DAPI, VAPB, and PTPIP51 on DIV26-30 OPCs generated from *FUS*^*P525L*^, *FUS*^*R521H*^ and their isogenic control hiPSCs (scale bar: 10 μm). **b**, **d** Violin plot of the intensity of VAPB staining on *FUS*^*P525L*^ and *FUS*^*R521H*^ OPCs, and their isogenic controls. Each symbol represents the mean intensity of VAPB from an image. Data are represented as mean ± SD from 3 biological (*N* = 30 images). **c**, **e** Violin plot of intensity of PTPIP51 staining on *FUS*^*P525L*^ and *FUS*^*R521H*^ OPCs, and their isogenic controls. Each symbol represents the mean intensity of PTPIP51 from an image. Data are represented as mean ± SD from three biological (*N* = 30 images). **f** Proximity ligation analysis (PLA) using antibodies against VAPB and PTPIP51 (VAPB/PTPIP51, red) *FUS*^*P525L*^ and *FUS*^*R521H*^ OPCs, and their isogenic controls. Nuclei were stained with DAPI (blue). Scale bars are 10 μm. **g**, **h** Violin plot of quantification of the PLA signal in *FUS*^*R521H*^ and isogenic control OPCs (**h**) and *FUS*^*P525L*^ and isogenic control OPCs (**i**). Each symbol represents the mean number of PLA dots per cell from one image. Statistical analyses were performed by unpaired two-tailed *t* tests to compare mutant *FUS* OPCs and their controls. Data are represented as mean ± SD from three biological replicates with three technical replicates in each experiment (*N* = 20 images and n > 100 nuclei/condition). **p < 0.01

Previous work suggested that the MAM is a central hub for lipid metabolism and regulation of mitochondrial function [3]. The lipidomic results demonstrated a decrease in PC/PE ratio in *FUS*^*R521H*^ OPCs compared to isogenic control. This would be consistent with a disruption of MAM, which has been reported to be a causal mechanism of neurodegenerative diseases [55], including ALS [69]. Therefore, PLA using antibodies against VAPB and PTPIP51 was used to quantitatively assess MAM. VAPB is an integral ER protein that interacts with protein tyrosine phosphatase-interacting PTPIP51, which is exclusively present in the outer mitochondrial membrane [102]. VAPB-PTPIP51 functions as a MAM tether, and their interactions can be used to monitor MAM formation in cells. First, we validated that the mRNA levels of *VAPB* and *PTPIP51* were not different in mutant *FUS* OPCs compared to their isogenic controls (Supplementary Fig. 8). In addition, we performed immunofluorescence staining of OPCs using anti-VAPB and anti-PTPIP51 antibodies (Fig. 6a). There was also no difference in VAPB and PTPIP51 protein levels between mutant *FUS* OPCs and their isogenic controls (Fig. 6b–e). This finding ensured that potential differences in PLA signals were not due to changes in the respective protein levels. However, the proximity ligation assay demonstrated that the number of VAPB-PTPIP51 puncta was severely reduced in mutant *FUS* OPCs compared to their isogenic controls (Fig. 6f–h), demonstrating that the number of ER-mitochondrial contact sites was reduced.

### Increased UPR activation in response to ER stress inducers in mutant *FUS* OPCs

**Fig. 7:**
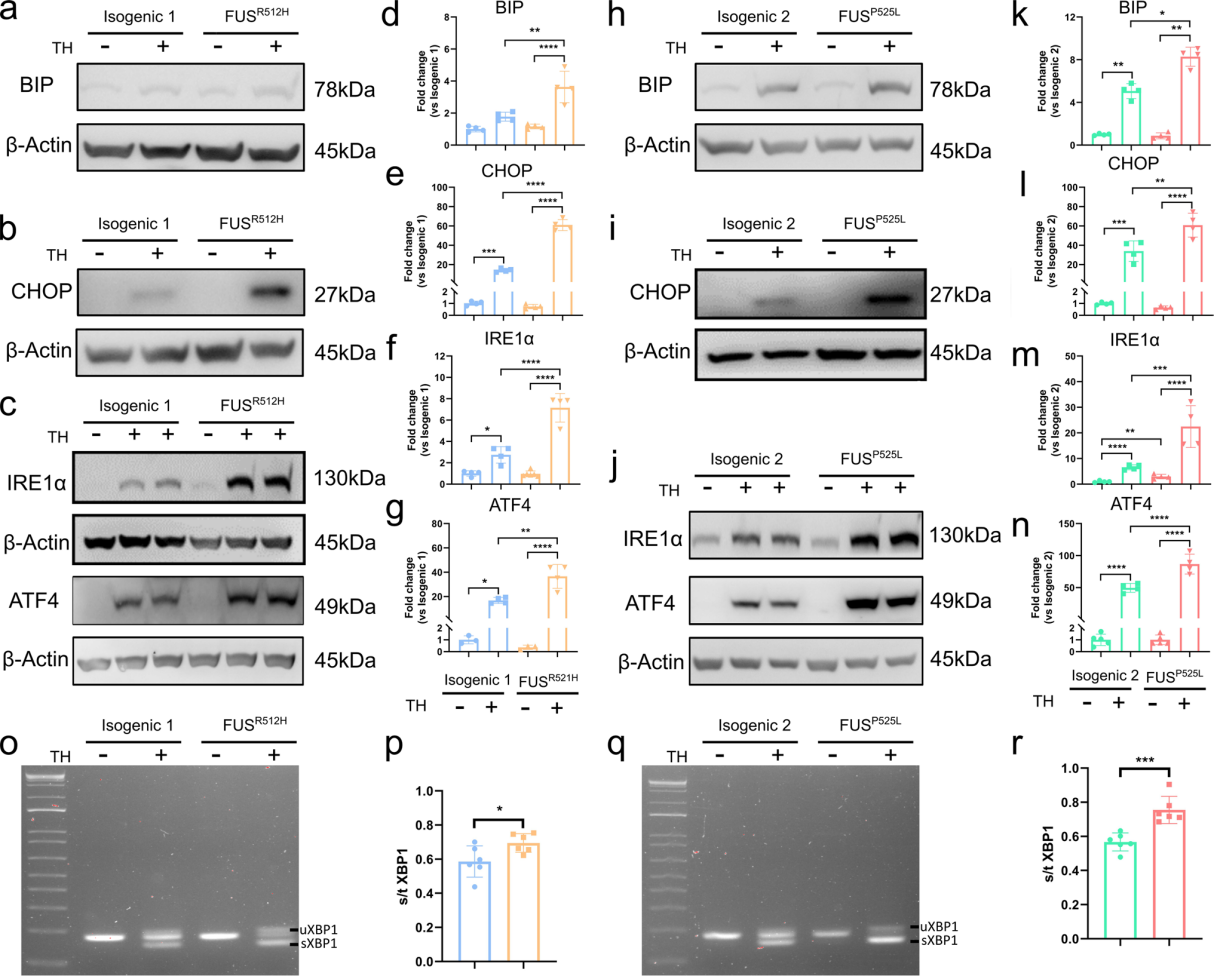
Mutant *FUS* iPSC-derived OPCs show enhanced ER stress after thapsigargin (TH) exposure. **a–g** Representative Western blots and quantification for BIP, CHOP, IRE1α, and ATF-4 levels in *FUS*^*R521H*^ and its isogenic control OPCs after 1 μM TH treatment for 24 h. All of the values are normalized to the control group (*N* ≥ 4 independent differentiations). **h–n** Representative Western blots of and quantification for BIP, CHOP, IRE1α, and ATF-4 levels in *FUS*^*P525L*^ and isogenic control OPCs after 1 μM TH treatment for 24 h (*N* ≥ 4 independent differentiations). **o**, **q** The mRNAs of spliced (sXBP1) and unspliced (uXBP1) were detected using RT-PCR in *FUS*^*R521H*^ and isogenic control OPCs (**o**) and *FUS*^*P525L*^ and isogenic control OPCs (**q**) after 2 μM TH treatment for 4 h. **p**, **r** Ratio of spliced to total *XBP1* mRNA in *FUS*^*R521H*^ and isogenic control OPCs (*N* = 6) and *FUS*^*P525L*^ and isogenic control OPCs (*N* = 6). Statistical analyses were performed by one-way ANOVA with the Bonferroni’s multiple comparisons test (**b–g** and **k–n**) and unpaired two-tailed *t* test (**p** and **r**) to compare *FUS*-mutant OPCs and its control. Data are represented as mean ± SD. *p < 0.05 **p < 0.01 ***p < 0.001 ****p < 0.0001

As MAM disruption is associated with ER stress, we monitored the levels of several markers of the unfolded protein response (UPR), a cellular program activated by ER stress, using Western blot analysis. We assessed the protein levels of BIP, CHOP, IRE1α, and ATF4 in mutant *FUS* and isogenic OPCs without and with triggering the cells with the ER stress inducer, thapsigargin (TH). TH causes ER Ca^2+^ depletion by blocking the sarco/endoplasmic reticulum Ca^2+^ ATPase (SERCA) [11]. ER Ca^2+^ depletion impairs proper chaperone functioning, thus resulting in the accumulation of unfolded proteins and subsequent ER stress [61]. At baseline, protein levels of BIP, CHOP, and ATF4 were not different between mutant *FUS* and isogenic control OPCs, even if a significant increase in IRE1α protein level was observed in *FUS*^*P525L*^ OPCs (Fig. 7j, m), but not in *FUS*^*R521H*^ OPCs (Fig. 7c, f). Following exposure to 1 μM TH for 24 h, protein levels of BIP, CHOP, IRE1α, and ATF4 were significantly increased in mutant *FUS* OPCs, compared to controls (Fig. 7a–g, h–n). In addition, we performed RT-qPCR to evaluate UPR after 2 μM TH treatment for 4 h, *CHOP*, *XBP1*, *PERK*, and *IRE1α* expressions increased significantly in mutant *FUS*, compared to their respective control OPCs (Supplementary Fig. 9). We also assessed the splicing of the *XBP1* transcript, which occurs as a result of activated IRE1α. We observed a significant increase in the spliced *XBP1* over total *XBP1* mRNA ratio in mutant *FUS* (Fig. 7o–r) *versus* isogenic control OPCs treated with 2 μM TH for 4 h. Altogether, these results indicate an increased UPR activation upon ER stress induction in *FUS*^*R521H*^ and *FUS*^*P525L*^ OPCs, compared to the isogenic controls, indicating increased susceptibility to ER stress with increased activation of pro-apoptotic ATF4/CHOP signaling.

### Impaired mitochondrial respiration in mutant *FUS* OPCs

**Fig. 8:**
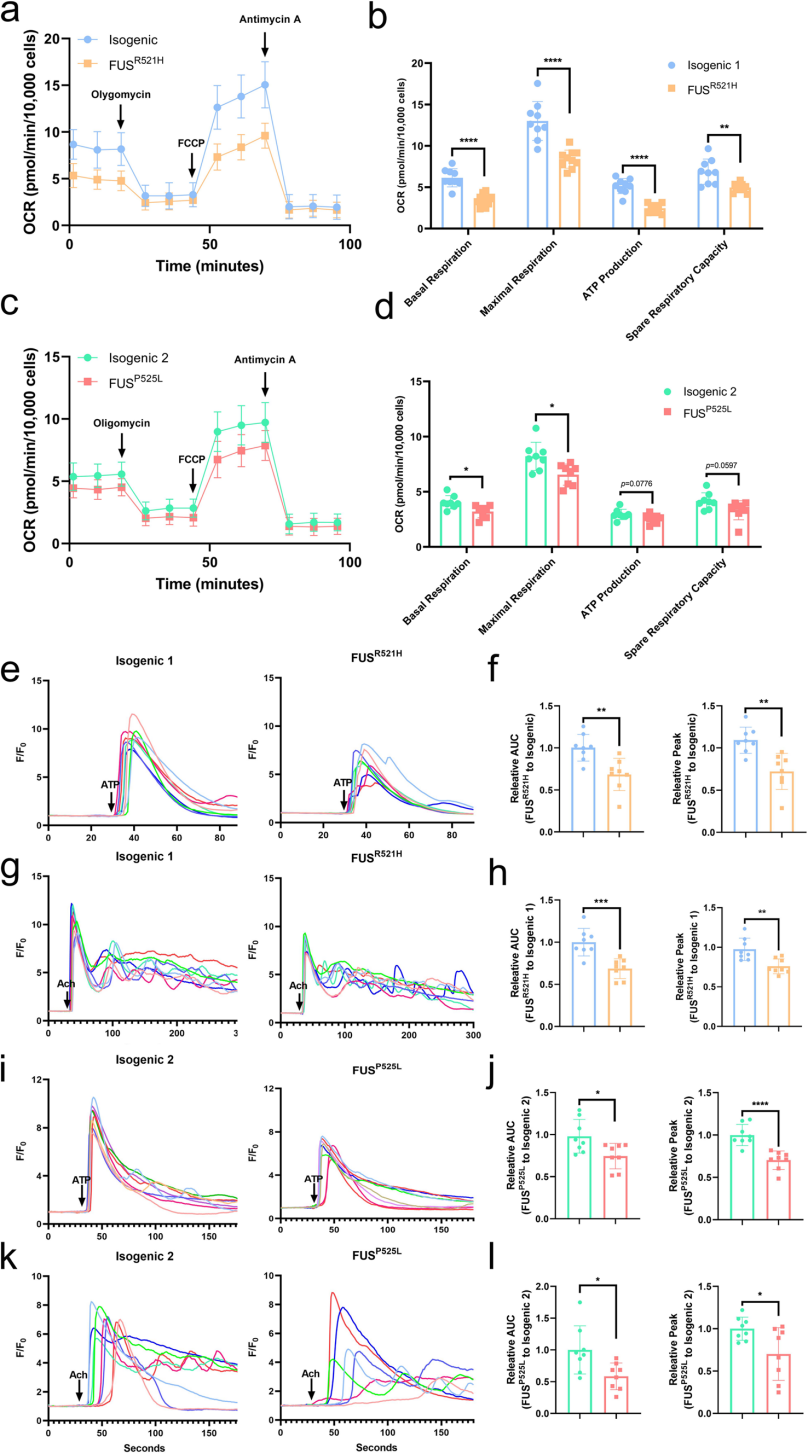
Mitochondrial respiration and ER-derived Ca^2+^ signaling are impaired in mutant *FUS* iPSC-derived OPCs. **a**, **c** Oxygen consumption rate (OCR) throughout the mitochondrial respiration in *FUS*^*R521H*^ OPCs (**a**) and *FUS*^*P525L*^ OPCs (**c**) and their respective isogenic controls (*N* ≥ 8). The time points of adding mitochondrial inhibitors to the media for evaluating respiratory parameters are indicated by arrows. **b**, **d** Basal respiration, maximal respiration, ATP production, and spare respiratory capacity in *FUS*^*R521H*^ OPCs (**b**) and *FUS*^*P525L*^ OPCs (**d**) and their respective isogenic controls. **e**, **i** Representative Ca^2+^ traces following 10 μM ATP stimulation (arrow) in *FUS*^*R521H*^ OPCs (**e**) and *FUS*^*P525L*^ OPCs (**i**) and their respective isogenic controls loaded with Cal-520. **f**,** h** Quantitative data of the corresponding AUC and peak after 10 μM ATP stimulation (*N* = 8). **g**, **k** Representative Ca^2+^ traces following 10 μM Ach stimulation (arrow) in *FUS*^*R521H*^ OPCs (**g**) and *FUS*^*P525L*^ OPCs (**k**) and their respective isogenic controls. **h**, **l** Quantitative data of the corresponding AUC and peak after 10 μM Ach stimulation (*N* = 8). Statistical analyses were performed by unpaired two-tailed* t* tests to compare mutant *FUS* OPCs and their control. Data are represented as mean ± SD. *p < 0.05 **p < 0.01 ***p < 0.001 ****p < 0.0001

To assess mitochondrial function, we performed Seahorse analysis on both pairs of mutant *FUS* and isogenic OPCs. This demonstrated that the basal respiration and maximal (uncoupled) respiration were significantly lower in mutant *FUS* OPCs compared to the isogenic controls (Fig. 8a–d). In addition, we noticed a significant decrease in ATP production and decreased spare respiratory capacity in mutant *FUS*^*R521H*^ OPCs compared to controls. Similarly, there was a diminished tendency in the mutant *FUS*^*P525L*^ OPCs (Fig. 8b, d).

### Decreased inositol 1,4,5-trisphosphate (IP_3_) receptor (IP_3_R)-mediated Ca^2+^ release in mutant *FUS* OPCs, compared to its control

Mitochondrial Ca^2+^ levels are critical to maintain proper mitochondrial energy production. The MAM plays a crucial role in the transfer of Ca^2+^ from ER to mitochondria [98]. At the MAM, IP_3_R channels are responsible for ER Ca^2+^ release, thus regulating the mitochondrial [Ca^2+^] [55]. Defects in IP_3_R-mediated Ca^2+^ release will, thus, impact mitochondrial Ca^2+^ levels and subsequent mitochondrial function. To determine whether IP_3_R function was affected in mutant *FUS* OPCs, we performed live cell Ca^2+^ imaging in Cal-520-loaded mutant *FUS* OPCs and isogenic controls. Therefore, we measured single-cell Ca^2+^ signals evoked by ATP and acetylcholine (Ach), both IP_3_-producing agonists, and compared Ca^2+^ responses between mutant *FUS* OPCs and isogenic controls. First, ATP (10 μM) was applied to activate cell-surface purinergic receptors and induce subsequent IP_3_ production and Ca^2+^ release [40]. Representative fluorescence traces illustrating ATP responses in OPCs are shown in Fig. 8e, i. We quantified fluorescence signals as a ratio (*F*/*F*_0_), and determined corresponding area under the curve (AUC) and peak amplitudes (*F*_peak_–*F*_baseline_). Both AUC and peak amplitude were lower in mutant *FUS* OPCs compared to those of its isogenic control (Fig. 8f, j). In addition to ATP (which may also activate ionotropic receptors and thus evoke Ca^2+^ influx), we also applied Ach as an agonist to trigger IP_3_R-medaited Ca^2+^ release [103]. Similarly, to the observations made for ATP, the AUC and the peak amplitude of the Ach-induced Ca^2+^ responses in mutant *FUS* OPCs were reduced compared to those occurring in the isogenic controls (Fig. 8g, k, h, l). In summary, mutant *FUS* OPCs displayed a decrease in IP_3_R-mediated Ca^2+^ release compared to their isogenic controls.

## Discussion

In this study, we generated oligodendroglia derived from human iPSC with two different *FUS* mutations, as well as isogenic control cells to evaluate defects caused by mutant *FUS* in oligodendroglia. The presence of the *FUS* mutations did not affect the efficiency of differentiation of iPSCs to O4^+^ OPCs. As could be expected, the presence of mutations in the NLS of *FUS* resulted in partial relocation of the FUS protein into the cytoplasm. We identified lipid metabolism defects in mutant *FUS* OPCs at RNA and metabolite levels, which may adversely affect membrane integrity and function. Decreased MAM formation was observed using PLA with antibodies against the ER–mitochondrial tethers VAPB and PTPIP51, which translated at the functional level in mitochondrial dysfunctions in association with increased susceptibility to ER stress inducers and decreased IP_3_R-mediated Ca^2+^ release.

We used two pairs of human iPSCs. One pair consisted of iPSCs derived from an ALS patient with a *FUS*^*R521H*^ mutation and its isogenic control. The second pair was created by knocking in the *FUS*^*P525L*^ mutation in a normal donor iPSC line. All cell lines were successfully differentiated into O4^+^ OPCs using SOX10 overexpression. We previously reported that O4^+^ cells generated by inducible SOX10 overexpression are transcriptionally similar to intermediate oligodendrocytes when compared with the transcriptome of human brain oligodendroglia [22], but not yet with fully mature oligodendrocytes. Nevertheless, we showed that the oligodendroglia generated from iSOX10 iPSCs could myelinate (albeit to a limited degree) neurons in vitro [22] and robustly myelinate neurons in vivo [21], and hence display mature oligodendroglial functions. Therefore, they are suitable for evaluating defects caused by mutations, such as the presence of mutations in *FUS*.

Mislocalization of FUS protein from the nucleus to the cytoplasm is a potent molecular hallmark of *FUS*-ALS pathology [27, 96] and most likely increases the vulnerability of neuronal cells both via a loss- and a gain-of-function mechanism [41]. We demonstrated that FUS mislocalizes in the cytoplasm of mutant OPCs, consistent with previous reports on oligodendrocytes in FUS mouse model and *FUS*-ALS patients [58, 82, 90]. Recent studies found that mutant *FUS* autoregulatory mechanisms are altered and contribute to its cytoplasmic accumulation [33, 81]. Consistent with the immunofluorescence data, a significant decrease in intron 6 and 7 retention was identified in OPCs with *FUS* NLS mutations. This decrease could lead to a vicious cycle of ever-increasing FUS protein in the cytoplasm, which may exert toxic effects. As a first screen to identify possible defects linked to mutant *FUS* in OPCs, we performed RNA sequencing experiment. This revealed ‘myelin sheath’ as one of the major upregulated pathways in both mutant *FU*S OPC populations, with significantly higher levels of different myelin-associated genes in the mutant compared to the isogenic control OPCs. By contrast, experiments performed in *FUS*-ALS mouse models demonstrated decreased expression of most crucial myelin-associated genes, such as *Myocilin*, *Ncmap*, *Pmp2*, *Pmp22*, *Cldn19,* and *Prx* in the spinal cords of knock-in *FUS* mice (*Fus*^*ΔNLS*^) [82]*.* A similar decrease in myelin sheath transcripts such as *Mbp* and *Plp1* was observed in the spinal cord of *FUS*^+*/*+^ mice [79], where presence of an extra copy of human FUS induces an ALS phenotype with death of animals around 60 days.

Due to the rarity of *FUS* mutations in ALS, transcriptomic studies are still lacking. Therefore, we evaluated RNAseq datasets of patients with other forms of ALS, i.e., ALS caused by the most frequent *C9orf72* mutation [50], and sporadic ALS [91] to assess a possible defect in myelin transcriptomic signature. Single nuclei RNAseq of OPC/oligodendrocytes from *C9orf72*-ALS motor cortex revealed a consistent pattern of downregulated genes associated with the ‘myelin sheath’. By contrast, analysis of published motor cortex RNAseq studies from patients with sporadic ALS [91] demonstrated elevated expression of *MBP* and *PLP1* compared to non-ALS controls. The divergence between *FUS*-mutant iPSC-derived OPCs and bulk mouse spinal cord extracts/human motor cortex remains elusive, and might arise from different pathological pathways occurring in these very distinct CNS regions, or the degree of disease progression.

Lipids play a critical role in myelin membranes, contributing to their stability and integrity. Lipid metabolism defects are recognized as a contributor to the pathological mechanism in ALS [23, 47]. Causal mutations in the gene coding for the enzyme SPTLC1 involved in lipid metabolism have been recently identified in juvenile ALS patients [35, 62], and TDP-43 is known to regulate transcription factors controlling lipid metabolism, especially cholesterol homeostasis [18, 30]. Furthermore, a growing number of recent transcriptomic and lipidomic analyses described altered lipid metabolism in the spinal cord of ALS mouse models, including *FUS*^+*/*+^ mice [8, 20, 79]. In ALS patients, variations of lipid levels such as glycerophospholipids correlate with disease progression [85]. Consistent with these data, our joint-pathway analysis of lipidomic and transcriptomic data from mutant *FUS* OPCs identified decreased levels of myelin-associated lipids, especially glycerophospholipids including PC and PI.

FUS, recognized as an RNA-binding protein, primarily governs gene expression by modulating post-transcriptional RNA control, including mRNA stabilization, localization, translation, and splicing [96]. To address the question of whether FUS can directly impact lipid metabolism, we explored in detail the genes targeted and spliced by FUS. We demonstrated that among 429 lipid metabolism-related genes, 130 genes are direct FUS targets or spliced by FUS. Furthermore, we observed that FUS is involved in almost all steps of the glycerophospholipid metabolism (Fig. 5n), the major lipid pathway dysregulated in mutant *FUS* OPCs. Consistently, we identified a number of lipid metabolism genes that can be regulated by FUS to be deregulated in both mutant iPSC-OPC populations. Analysis of mutant *FUS* mice spinal cord RNAseq studies identified an even greater number of lipid genes, and lipid gens that can be regulated by FUS to be aberrantly expressed, supporting the notion that FUS may be involved in the lipid defect. However, we also identified deregulated lipid genes in oligodendroglia and OPCs from *C9orf72*-ALS patient and in the motor cortex of a subgroup of sporadic ALS patient, including a limited number of lipid genes that can be regulated by FUS. As TDP-43, often mislocalized in sporadic ALS [89] and dipeptide repeats created by the *C9orf72* mutation [105], are also RNA-binding proteins, the underlying pathology in mutant *FUS*, *C9orf72* and sporadic ALS may share some similarities. Indeed, the dysfunctional splicing activity of TDP-43 in *SOD1*^*G93A*^ mice has been shown to trigger alterations in lipid-related genes [93]. These observations underscore the pivotal role played by RNA-binding proteins in the control of lipid metabolism. Thus, there is a possibility that FUS may be involved in the deregulated abnormal lipid metabolism in oligodendroglia, even if it is difficult to state this unequivocally, as deregulation of lipid metabolism genes was also observed in the motor cortex of a subpopulation of patients with sporadic ALS and in OPCs and oligodendrocytes of patients with *C9orf72*-mutant ALS.

Glycerophospholipid composition profoundly influences cellular processes, particularly in the membranes of mitochondria and ER [97]. The ER–mitochondria contact sites (or MAM) facilitate glycerophospholipid trafficking, with PC being synthesized from PS via PE [99]. To maintain membrane integrity, PC synthesized in the ER must eventually be translocated to mitochondria. In mammals, the majority of glycerophospholipids in mitochondria are initially generated in the ER, and lipid exchange occurs through the MAM [67]. There is mounting evidence that a decrease in PC (reflected in a decreased PC/PE ratio) is involved in neurodegeneration, including MN disease [27, 75]. In addition, free cholesterol can be converted to cholesteryl esters (CE) at MAM by the enzyme acyl CoA:cholesterol acyltransferase 1 [10]. In this study, we observed a decrease in the PC/PE ratio in *FUS*^*R521H*^ mutant OPCs, associated with decreased levels of cholesterol esters. For that reason, we also examined whether defects in MAM could be demonstrated in mutant *FUS* OPCs.

Causal mutations in proteins such as SIGMAR1 or VABP, both ER membrane-expressed proteins involved in MAM formation, have been identified in familial cases of juvenile ALS [1, 25, 51, 101, 104]. There is also evidence that mutant SOD1, TDP-43, or FUS proteins accumulate in ectopic compartments in ALS neural cells, where they can perturb MAM function [86, 87, 104]. Here, we demonstrated by PLA, that close interactions between the mitochondrial protein, PTPIP51 and the ER protein, VAPB, were significantly decreased in mutant *FUS* OPCs compared to control OPCs, consistent with decreased ER–mitochondrial contact sites in mutant *FUS* OPCs. A similar observation was previously made in mutant *FUS* MNs [27]. This is the first study to describe a MAM phenotype in ALS oligodendroglia. Interestingly, we could identify a similar MAM phenotype in both pairs of mutant *FUS* OPCs, regardless of whether they were derived from a patient with a mutation in *FUS* or generated through knocking in a *FUS* mutation. This indicates that the mutant *FUS* itself is sufficient to cause the observed MAM defect.

Disruptions in MAM function have been associated with ER and/or mitochondrial stress, and with perturbations in Ca^2+^ homeostasis [7, 13, 55]. In particular, mitochondrial metabolism is dependent on ER–mitochondrial contacts, e.g., for the delivery of Ca^2+^ from the ER toward the mitochondria [9, 77]. Therefore, defects in MAM also diminish mitochondrial metabolism and contribute to ALS pathogenesis [2]. This was exactly what was observed here as mitochondrial respiration in OPCs derived from mutant *FUS* iPSCs was severely impaired. In addition, exposure of mutant *FUS* OPCs to TH, a SERCA inhibitor that evokes ER stress by depleting ER Ca^2+^ stores resulted in a profound activation of the UPR pathway, suggesting increased susceptibility to ER stress inducers. TH evoked a stronger increase in BIP, a major ER chaperone, as well as CHOP, a protein involved in apoptosis, in mutant *FUS* OPCs compared to its isogenic controls. Western blotting also demonstrated a significantly larger induction of two of the three main arms of the UPR, increased IRE1α-XBP1 signal and ATF4 downstream target of the PERK pathway in response to TH in mutant *FUS* OPCs. Although we tested three different anti-ATF6 antibodies, to assess the third UPR pathway, we could not discriminate between full-length and spliced ATF6, and, hence, could not determine whether this pathway is also activated. Finally, we also demonstrated a decreased IP_3_R-mediated Ca^2+^ release in mutant *FUS* OPCs. Therefore, our studies suggest perturbed MAM function in *FUS*‑mutant OPCs with increased ER stress susceptibility and deranged Ca^2+^ signaling. Whether thapsigargin-induced ER stress would exacerbate the MAM or lipid metabolism defects might be interesting to evaluate.

Several limitations are notable within our study, primarily stemming from the unavailability of cerebral cortex or spinal cord tissues from patients with *FUS* mutations, thus impeding the validation of our findings. In addition, the causative relationship between lipid metabolism defects and the compromised function of cellular membranes, particularly the disruption of the MAM, remains ambiguous. The observation that FUS targets and splices many genes involved in lipid metabolism suggests a direct impact on lipid metabolism, with a subsequent effect on MAM. However, further investigations are essential to ascertain whether *FUS* mutations lead to direct alterations in FUS capacities to bind and splice lipid-linked genes, while the modulation of lipid metabolism will determine the possible reversion of MAM defects. Furthermore, the impact of the MAM dysfunction in mutant *FUS* OPCs on the overall oligodendroglial function, specifically with regard to myelination, is yet to be determined. Although alignment between MBP^+^ oligodendroglia and neuronal axons can be readily demonstrated in culture [22], few studies have succeeded in demonstrating robust in vitro axonal myelination by oligodendrocytes in hiPSC-based models. The functional study of myelination will require the transplantation of human iPCS-derived OPCs within mouse brain, as we previously described [21]. Another limitation of our study is that we only used a single mutant iPSC line for each of both mutations. It is well known that there is heterogeneity among lines from different donors and even within the same donor. Minor variations at each stage of iPSC culture and differentiation can cumulatively lead to significantly different outcomes [73]. In addition, genomic instability can occur at any iPSC generation stage, and mutations may arise during iPSC differentiation [52, 57].

In conclusion, our study provides, for the first time, evidence of MAM defects in oligodendroglia generated from iPSCs. These MAM defects are associated with aberrant lipid metabolism, ER stress, mitochondrial dysfunction, and decreased Ca^2+^ signaling. This discovery opens new avenues for understanding the role of mutant *FUS* in oligodendroglial function and its potential contribution to MN degeneration.

### Supplementary Information

Below is the link to the electronic supplementary material.

Supplementary file1 (TIFF 16436 KB)

Supplementary file2 (PDF 2298 KB)

## Data Availability

The datasets analyzed in the current study are available from the corresponding author on reasonable request.
