# Correction to: Disruption of MAM integrity in mutant *FUS* oligodendroglial progenitors from hiPSCs

**DOI:** 10.1007/s00401-024-02748-4

**Published:** 2024-06-13

**Authors:** Yingli Zhu, Thibaut Burg, Katrien Neyrinck, Tim Vervliet, Fatemeharefeh Nami, Ellen Vervoort, Karan Ahuja, Maria Livia Sassano, Yoke Chin Chai, Arun Kumar Tharkeshwar, Jonathan De Smedt, Haibo Hu, Geert Bultynck, Patrizia Agostinis, Johannes V. Swinnen, Ludo Van Den Bosch, Rodrigo Furtado Madeiro da Costa, Catherine Verfaillie

**Affiliations:** 1 Department of Development and Regeneration, Stem Cell Institute, KU Leuven, 3000 Leuven, Belgium; 2 Department of Neurosciences, Experimental Neurology, KU Leuven, Leuven Brain Institute (LBI), 3000 Leuven, Belgium; 3 Laboratory of Neurobiology, VIB, Center for Brain and Disease Research, 3000 Leuven, Belgium; 4 Laboratory of Molecular and Cellular Signalling, Department of Cellular and Molecular Medicine, KU Leuven, 3000 Leuven, Belgium; 5 Laboratory of Cell Death Research and Therapy, Department of Cellular and Molecular Medicine, KU Leuven, 3000 Leuven, Belgium; 6 Center for Cancer Biology, VIB, 3000 Leuven, Belgium; 7 Animal Physiology and Neurobiology Section, Department of Biology, Neural Circuit Development and Regeneration Research Group, 3000 Leuven, Belgium; 8 National Engineering Research Center for Modernization of Traditional Chinese Medicine-Hakka Medical Resources Branch, School of Pharmacy, Gannan Medical University, Ganzhou, China; 9 Laboratory of Lipid Metabolism and Cancer, Department of Oncology, KU Leuven, 3000 Leuven, Belgium

**Correction to: Acta Neuropathologica (2024) 147:6** 10.1007/s00401-023-02666-x

In Figure 5 of this article, the incorrect dataset has been used to compare *FUS*^*R521H*^ and Isogenic 1 OPCs. Hence, the data related to *FUS*^*R521H*^ OPC presented in Figure 5, panels l, m, and n are incorrect. The Fig. 5 (panels I, m and n) should have appeared as shown below.

**Fig. 5:**
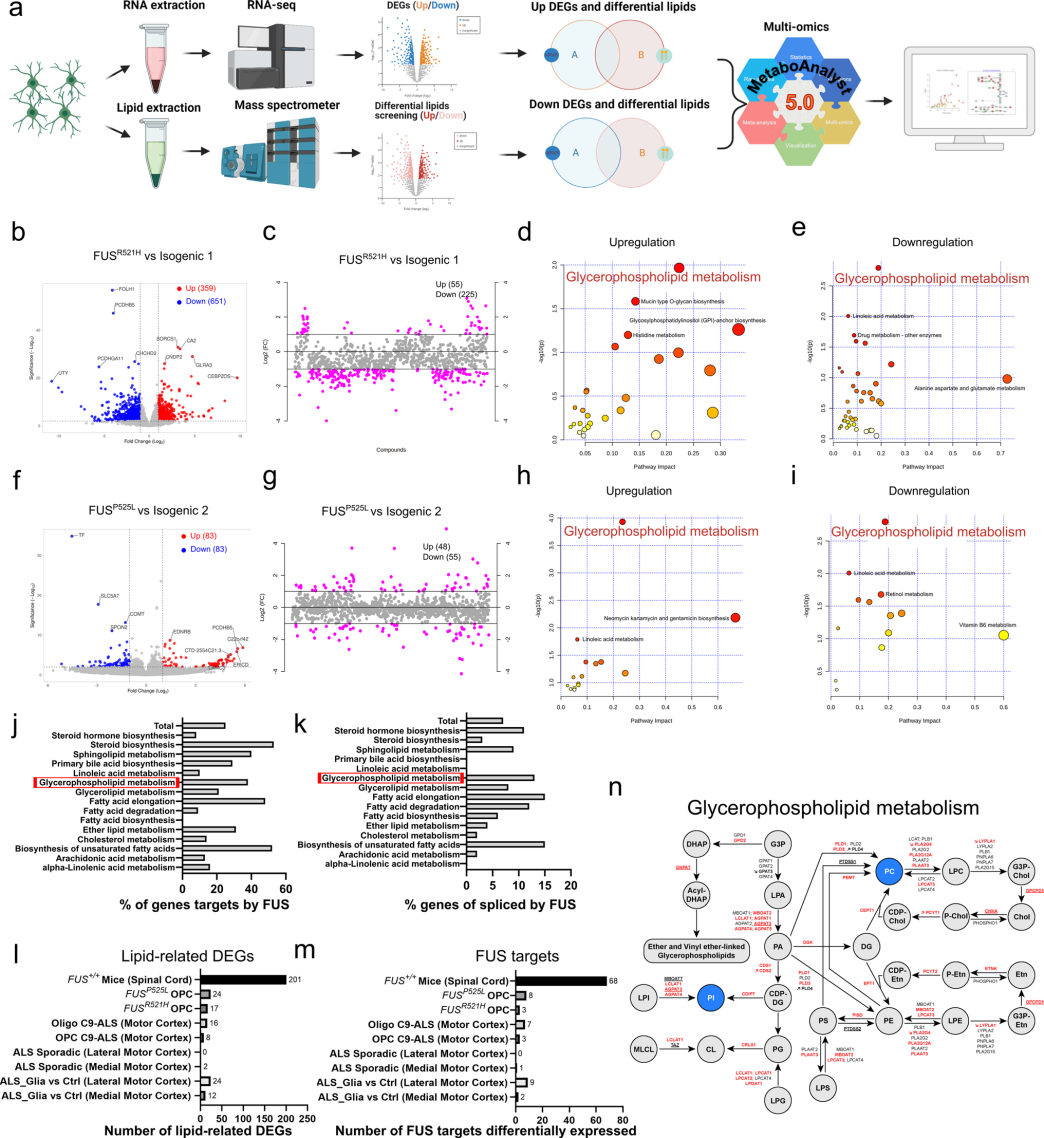
Dysregulated glycerophospholipid metabolism in mutant *FUS* iPSC-derived OPCs. **a** Scheme of joint-pathway analysis. **b**, **f** Volcano plots of upregulated (red) and downregulated (blue) genes in *FUS*-mutant OPCs, compared to isogenic controls. Genes with log_2_FC < − 1.0 and − log_10_(*p*) > 2.0 were considered downregulated and log_2_FC > 1.0 with − log_10_(*p*) > 2.0 were considered upregulated. **c**,** g** Important dysregulated lipid species (red circles) selected by fold-change analysis. Both upregulated (log_2_FC > 1.0) and downregulated (log_2_FC < − 1.0) features are plotted in a symmetrical way. **d**, **h**,** e**,** i** Joint-pathway analysis (MetaboAnalyst v.5.0) shows upregulated and downregulated metabolic pathways in *FUS*-mutant OPCs, compared to isogenic controls. Each circle signifies a distinct pathway, with its size and shade reflecting the pathway’s impact and statistical significance (red denotes the highest significance). **j**, **k** Bar graphs displaying the percentage of known genes targeted by FUS (**j**) or spliced by FUS (**k**) within the different lipid-metabolism-related KEGG pathways (based on studies [12, 33, 37, 45, 64, 83]. **l**, **m** Bar graphs indicating the number of lipid-metabolism-related dysregulated genes (DEGs) (**l**) and the number of lipid-metabolism-related dysregulated genes that can be regulated by FUS (**m**) across previously published datasets compared to *FUS*-mutant OPCs. These datasets include bulk RNAseq data of the spinal cord from symptomatic *FUS*^+*/*+^ mice overexpressing wild-type human FUS compared to wild-type mice [79], single nuclei RNAseq data of primary human motor cortex OPCs/oligodendroglia of *C9orf72*-ALS patients compared with control human brain [50] and bulk RNAseq data of sporadic ALS patient motor cortex compared to control non-ALS control individuals [91], as well as between the so-called motor cortex of a ‘ALS_glia’ subtype group (identified by enriched astroglia, microglia and oligodendroglia dysregulated genes, and this even if—according to the authors—there was no selective neuronal loss) versus motor cortex all other sporadic ALS patients. **n** Scheme of glycerophospholipid metabolism, in which circles indicate metabolites and arrows indicate the enzymatic reaction with the gene name encoding the enzyme. Altered lipid classes in *FUS*-mutant OPCs are highlighted in blue, and arrows indicate whether the gene is upregulated or downregulated based on RNAseq data. Genes targeted by FUS are highlighted in red, while genes spliced by FUS are underlined

In the sentence beginning “Furthermore, we found 89 and 24 lipid-related” in this article, the text “Furthermore, we found 89 and 24 lipid-related genes aberrantly expressed in *FUS*^*R521H*^ and *FUS*^*P525L*^ OPCs, respectively (Figure 5l). Importantly, 30 lipid metabolism genes that can be regulated by FUS were found aberrantly expressed in the mutant *FUS*^*R521H*^ OPCs and 8 in the mutant *FUS*^*P525L*^ OPCs (Figure 5m)” should have read “Furthermore, we found 17 and 24 lipid-related genes aberrantly expressed in *FUS*^*R521H*^ and *FUS*^*P525L*^ OPCs, respectively (Fig. 5l). Notably, 3 lipid metabolism genes that can be regulated by FUS were found aberrantly expressed in the mutant *FUS*^*R521H*^ OPCs and 8 in the mutant *FUS*^*P525L*^ OPCs (Fig. 5m)."

