# Supplementary figures and images for: Disruption of MAM integrity in mutant FUS oligodendroglial progenitors from hiPSCs
Source: Acta Neuropathol. 2024 Jan 3;147(1):6. doi: 10.1007/s00401-023-02666-x (PMC10764485; doi:10.1007/s00401-023-02666-x)

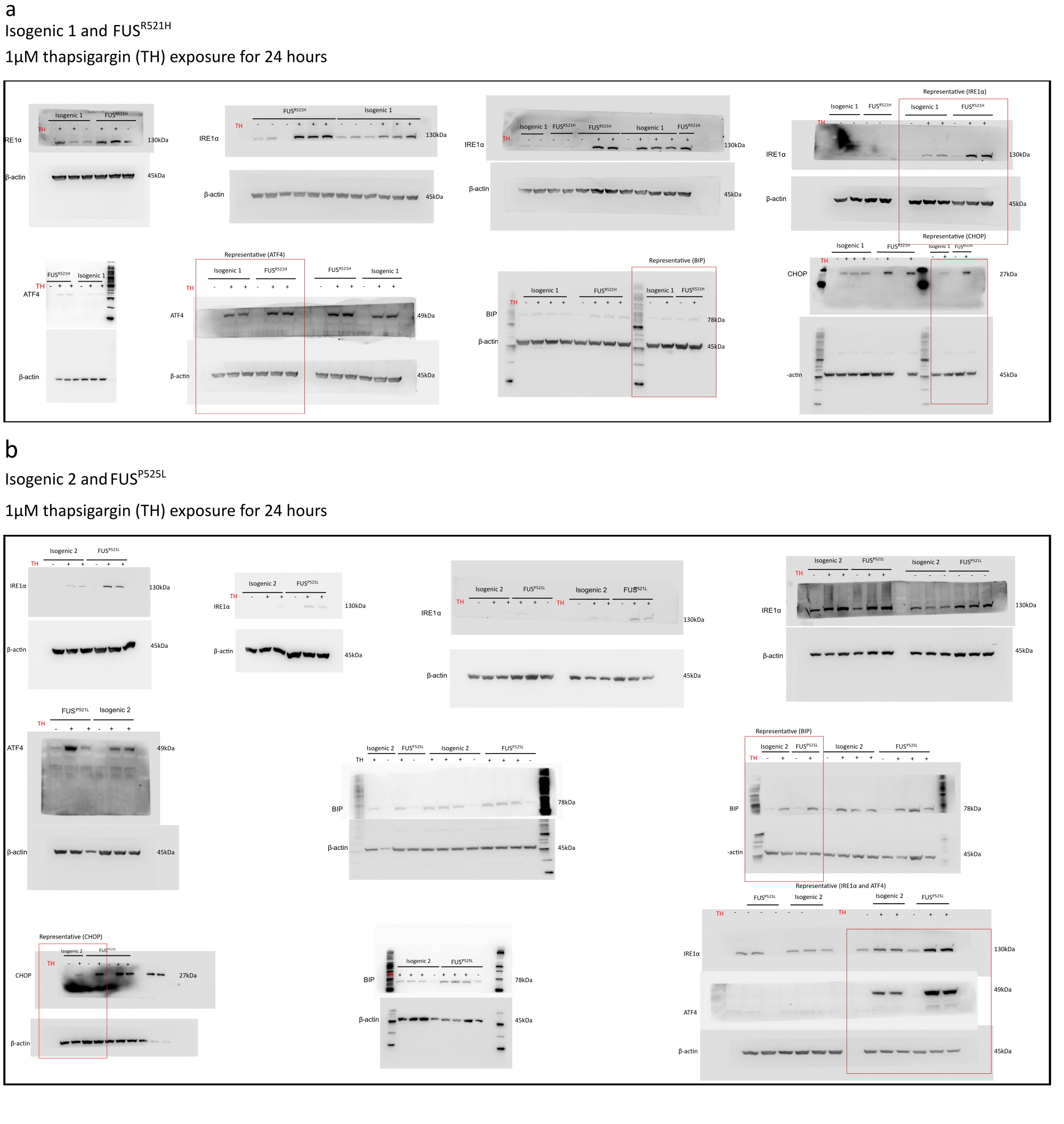

Supplement: Supplementary file 1 — Supplementary file1 (TIFF 16436 KB) [file 401_2023_2666_MOESM1_ESM.tiff]
