# Supplementary material for: Disruption of MAM integrity in mutant FUS oligodendroglial progenitors from hiPSCs
Source: Acta Neuropathol. 2024 Jan 3;147(1):6. doi: 10.1007/s00401-023-02666-x (PMC10764485; doi:10.1007/s00401-023-02666-x)
Supplement: Supplementary file 2 — Supplementary file2 (PDF 2298 KB) [file 401_2023_2666_MOESM2_ESM.pdf]

Supplemental Information

Disruption of MAM Integrity in Mutant *FUS* Oligodendroglial Progenitors from hiPSCs

Yingli Zhu[1+*], Thibaut Burg[2,3+], Katrien Neyrinck[1+], Tim Vervliet[4], Fatemeharefeh Nami[1], Ellen Vervoort[5,6], Karan Ahuja[1,7], Maria Livia Sassano[5,6], Yoke Chin Chai[1], Arun Kumar Tharkeshwar[2,3], Jonathan De Smedt[1], Haibo Hu[8], Geert Bultynck[4], Patrizia Agostinis[5,6], Johannes V. Swinnen[9], Ludo Van Den Bosch[2,3], Rodrigo Furtado Madeiro da Costa[1], Catherine Verfaillie[1]

[1]Stem Cell Institute, Department of Development and Regeneration, KU Leuven, 3000 Leuven, Belgium
[2]KU Leuven, Department of Neurosciences, Experimental Neurology and Leuven Brain Institute (LBI), 3000 Leuven, Belgium.
[3]VIB, Center for Brain & Disease Research, Laboratory of Neurobiology, Leuven, 3000, Belgium.
[4]KU Leuven, Laboratory of Molecular and Cellular Signalling, Department of Cellular and Molecular Medicine, 3000 Leuven, Belgium
[5]Laboratory of Cell Death Research & Therapy, Department of Cellular and Molecular Medicine, KU Leuven, 3000 Leuven, Belgium
[6]VIB, Center for Cancer Biology, 3000 Leuven, Belgium
[7]Neural Circuit Development and Regeneration Research Group, Animal Physiology and Neurobiology Section, Department of Biology, 3000 Leuven, Belgium
[8]National Engineering Research Center for Modernization of Traditional Chinese Medicine - Hakka Medical Resources Branch, School of Pharmacy, Gannan Medical University, Ganzhou, China
[9]Laboratory of Lipid Metabolism and Cancer, Department of Oncology, KU Leuven, 3000 Leuven, Belgium

[*]Correspondence: yingli.zhu@kuleuven.be; Telephone: +32 487787593

[+]These authors contributed equally to this work.

Supplementary Fig. 1

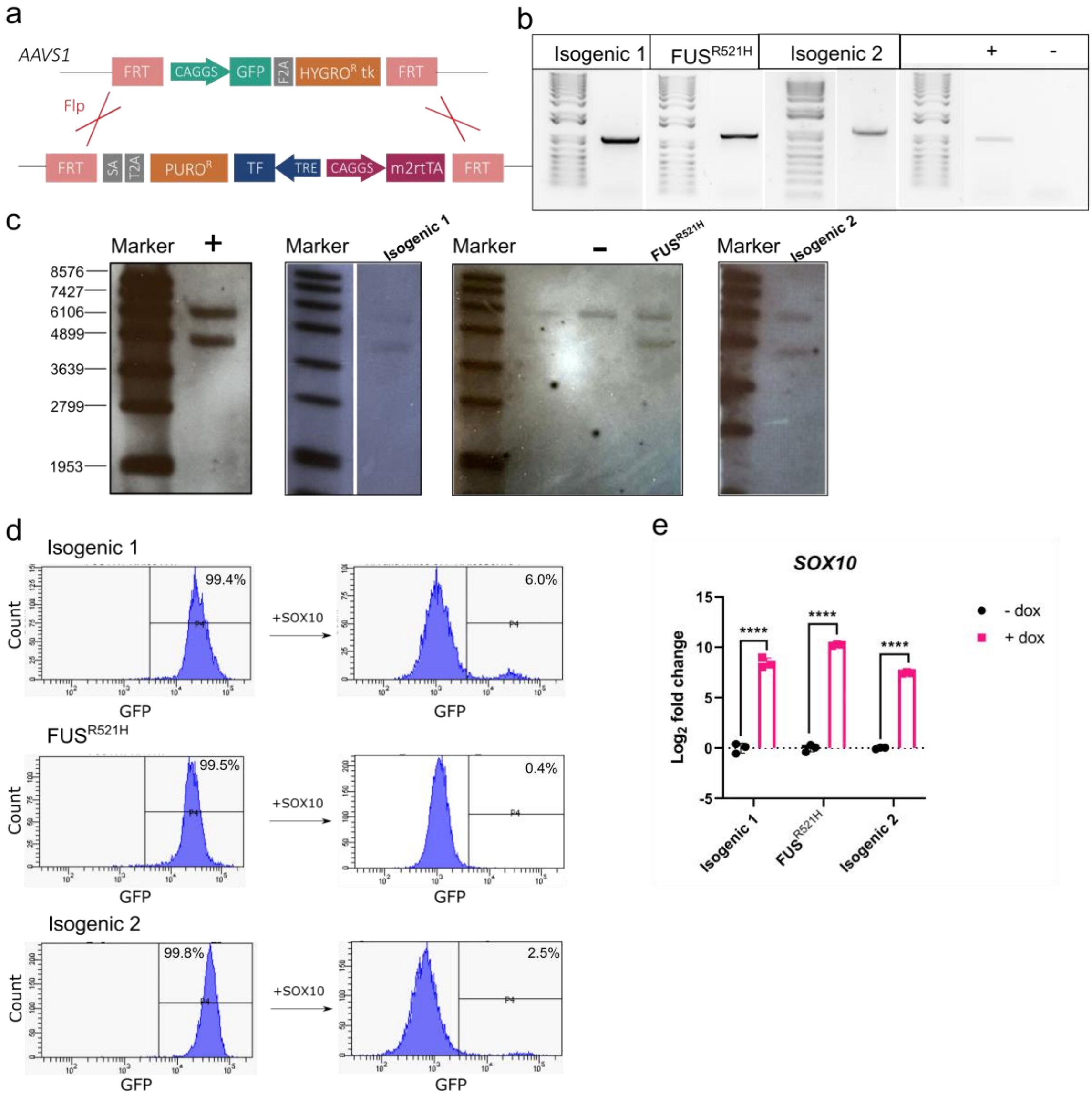

Supplementary Fig. 1 Integration of inducible SOX10 inside *FUS^R521H* mutant and its isogenic control iPSC as well as SIGi001-A iPSC via RMCE.

a Overview of the recombinase-mediated cassette exchange (RMCE) process. First, a master cell line was created by inserting a GFP-Hygro-TK cassette flanked by FRT-sites inside the *AAVS1* locus with the use of ZFNs. Next, via RMCE this GFP-Hygro-TK cassette was exchanged for the cassette containing the TF *SOX10* under a TET-ON promotor and a puromycin selection gene, which was again flanked by FRT-sites. **b** 5' junction PCR on the genomic DNA of the master cell lines with one primer outside and one primer inside the GFP-Hygro-TK cassette. **c** Southern blot on the genomic DNA, cleaved by NcoI, of the master cell lines using a probe recognizing the homology arm to detect possible random integration events. **d** Flow cytometry demonstrating excision of the GFP-containing cassette after RMCE. **e** RT-qPCR of *SOX10* with and without the addition of doxycycline to the genome-engineered iPSC lines (*N*=3 independent differentiations). Statistical analyses were performed by unpaired two-tailed *t*-tests to compare mutant *FUS* and its control. Data are represented as mean ± SD. $****p<0.0001$.

Supplementary Fig. 2

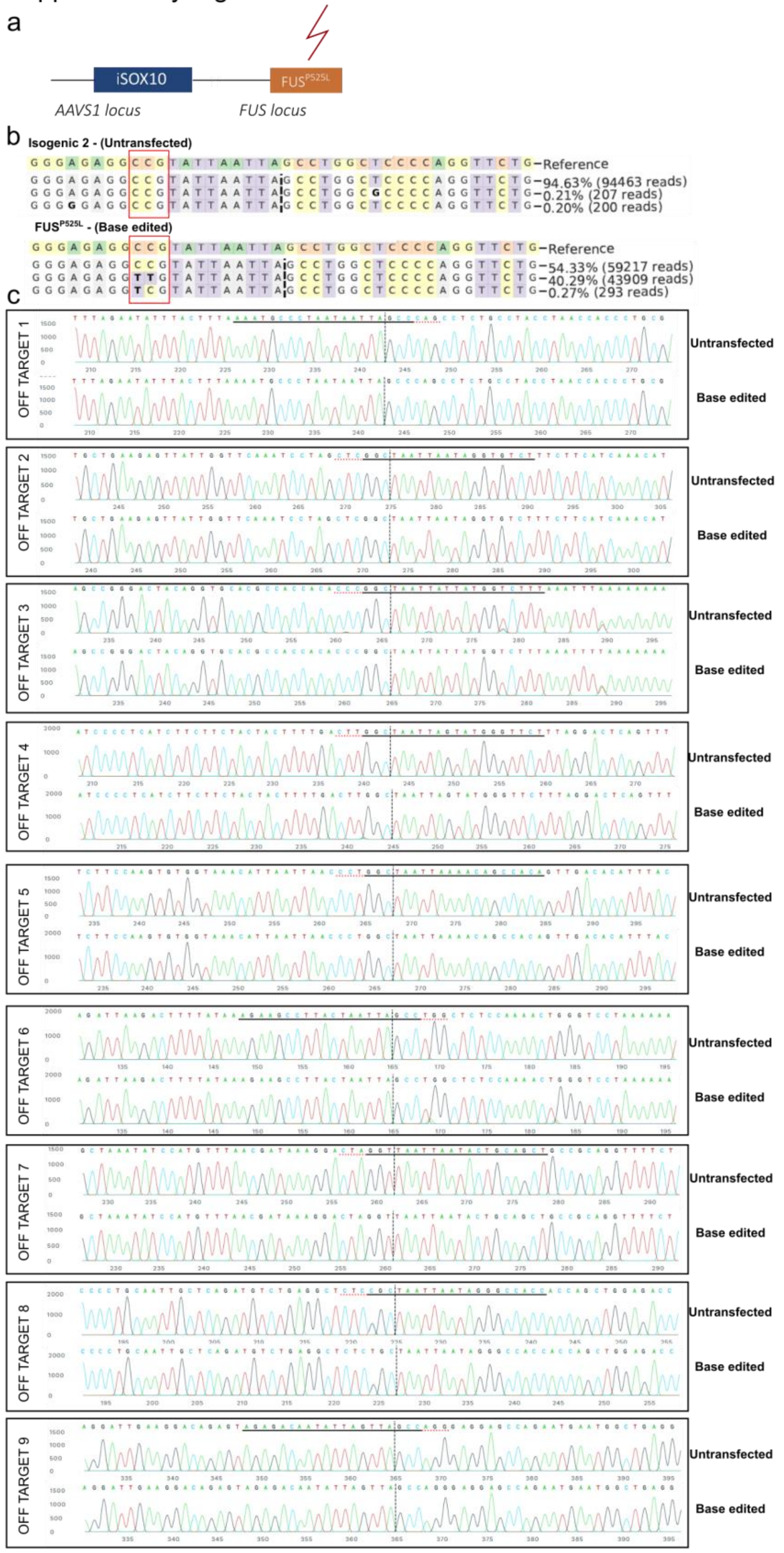

Supplementary Fig. 2 Insertion of a *FUS*^{P525L} mutation in the iSOX10 normal donor SIGi001-A iPSC line via base editing.

a Via base editing, a *FUS*^{P525L} mutation is created in the endogenous *FUS* locus of the SIGi001-A iPSC lines that already contain the *iSOX10* cassette in the *AAVS1* locus. **b** Sanger and Deep sequencing results of the *FUS* target locus before and after base editing in SIGi001-A iSOX10-iPSCs indicate insertion of the *P525L* mutation. **c** Sanger sequencing results of *in silico* predicted off-target regions show no off-target events.

Supplementary Fig. 3

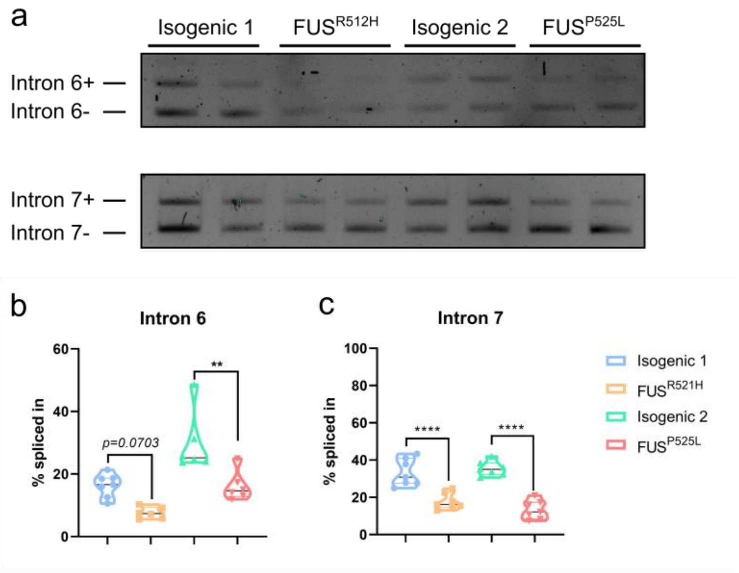

Supplementary Fig. 3 Intron 6 or 7 retention decreased in mutant *FUS* iPSC-derived OPCs.

a Representative gel electrophoresis of RT-PCR assays identifying RNA species with or without intron 6 retention, and with or without intron 7 retention in OPCs. **b, c** The percentage of intron 6 (**b**) or 7 (**c**) retention (intron + band intensity divided by the sum of intensities of intron + and intron – bands, multiplied by 100) ($N \geq 6$). Statistical analyses were performed by one-way ANOVA with the Bonferroni's multiple comparisons test to compare *FUS* mutant OPCs and its control. Data are represented as mean ± SD. **$p<0.01$ ****$p<0.0001$.

Supplementary Fig. 4

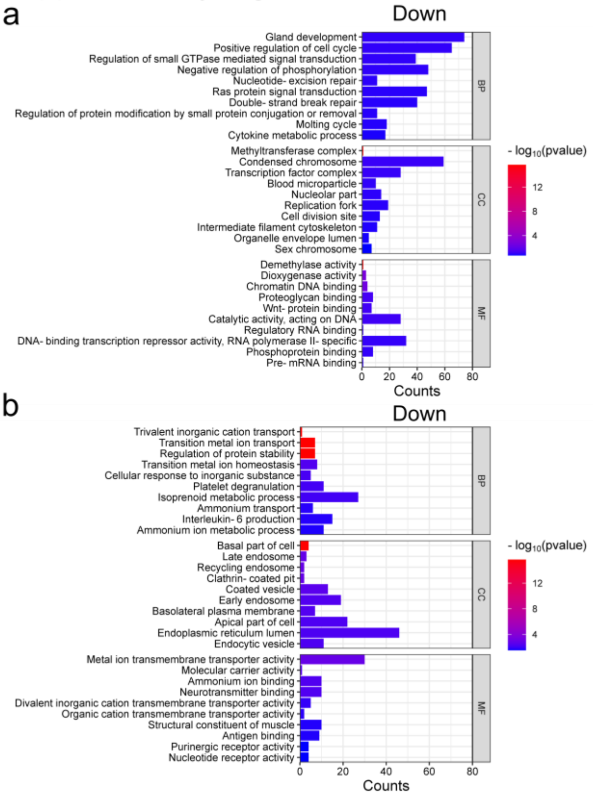

Supplementary Fig. 4 Transcriptome analysis of downregulated genes in mutant *FUS* OPCs.

a, b Ranked gene set enrichment analysis with Gene Ontology (GO) was performed in FUS^{R521H} mutant and its isogenic control OPCs (**a**) and FUS^{P525L} mutant and its isogenic control OPCs (**b**) using the online tool WebGestalt. The top 10 GO enrichment terms of downregulated genes in biological process (BP) ontology, cellular component (CC) ontology, and molecular function (MF) ontology.

Supplementary Fig. 5

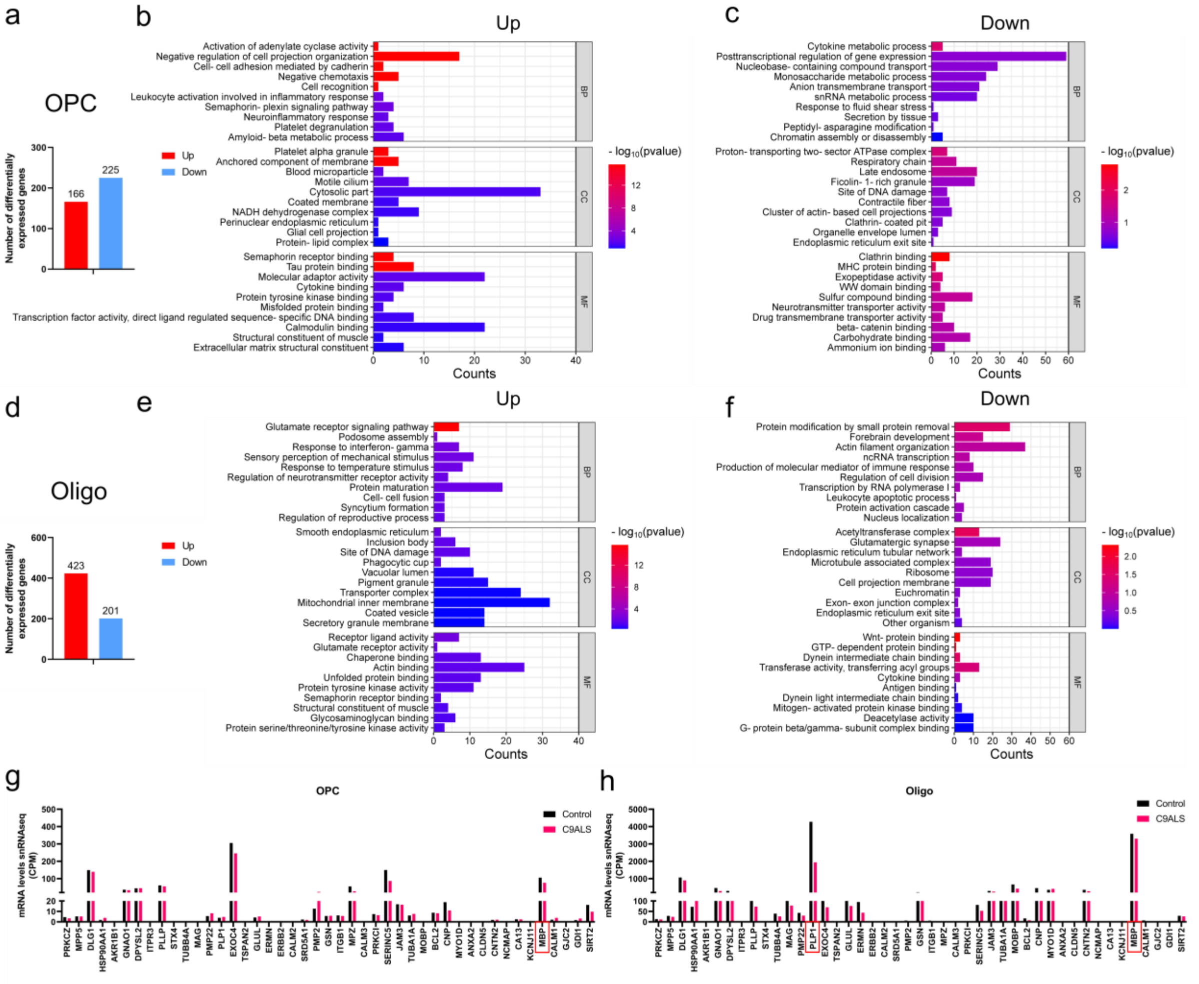

Supplementary Fig. 5 Single nuclei RNAseq analysis did not show myelin sheath signature in OPCs and Oligodendrocytes of Motor Cortex from *C9orf72*-ALS patients compared to healthy controls.

Single nuclei RNAseq (snRNA-seq) study of ALS patient motor cortex compared to healthy control was reanalyzed, with a focus on the OPCs and Oligodendroglia [4]. **a, d** Number of DEGs (Fold-change>1.2, FDR<0.05) in motor cortex OPCs (**a**) and oligodendrocytes (**d**) from *C9orf72*-ALS patients, compared to healthy controls, based on snRNA-seq data. **b, c** Bar plot displaying the 10 gene sets most significantly enriched among the up-regulated (**b**) and the down-regulated (**c**) pathways in motal cortex OPCs from *C9orf72*-ALS patients, compared to healthy controls, based on GSEA (minGSSize = 5; maxGSSize = 2000). **E, f** Bar plot displaying the 10 gene sets most significantly enriched among the up-regulated (**e**) and the down-regulated (**f**) pathways in motal cortex oligodendrocytes from *C9orf72*-ALS patients, compared to healthy controls, based on GSEA (minGSSize = 5; maxGSSize = 2000). **g, h** Normalized expression of myelin sheath-related genes in motor cortex OPCs (**g**) and oligodendrocytes (**h**) from *C9orf72*-ALS patients, compared to healthy controls, based on CPM from snRNAseq.

Supplementary Fig. 6

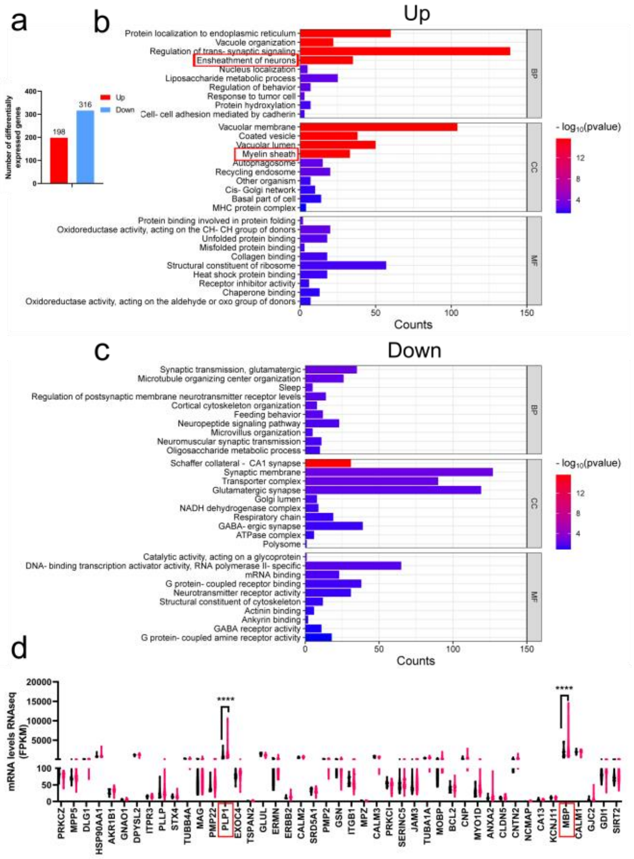

Supplementary Fig. 6 Transcriptome analysis showed myelin sheath signature in sporadic ALS patients' motor cortex, compared to healthy controls.

Bulk RNAseq study of the motor cortex from sporadic ALS patients compared to non-ALS control individuals was reanalyzed [8]. **a** The number of DEGs (Fold-change>2, FDR<0.1) in the motor cortex from sporadic ALS patients, compared to healthy controls. **b, c** Bar plot displaying the 10 gene sets most significantly enriched among the up-regulated (**b**) and the down-regulated (**c**) pathways in motor cortex OPCs from sporadic ALS patients, compared to healthy controls, based on GSEA (minGSSize = 5; maxGSSize = 2000). **d** Normalized expression of myelin sheath-related genes (*PLP1* and *MBP*) in the motor cortex in sporadic ALS patient samples against those from individuals without neurological disorders, based on FPKM from RNAseq. Statistical analyses were performed by two-way ANOVA with the Bonferroni's multiple comparisons test. Data are represented as mean ± SD. ****p<0.0001.

Supplementary Fig. 7

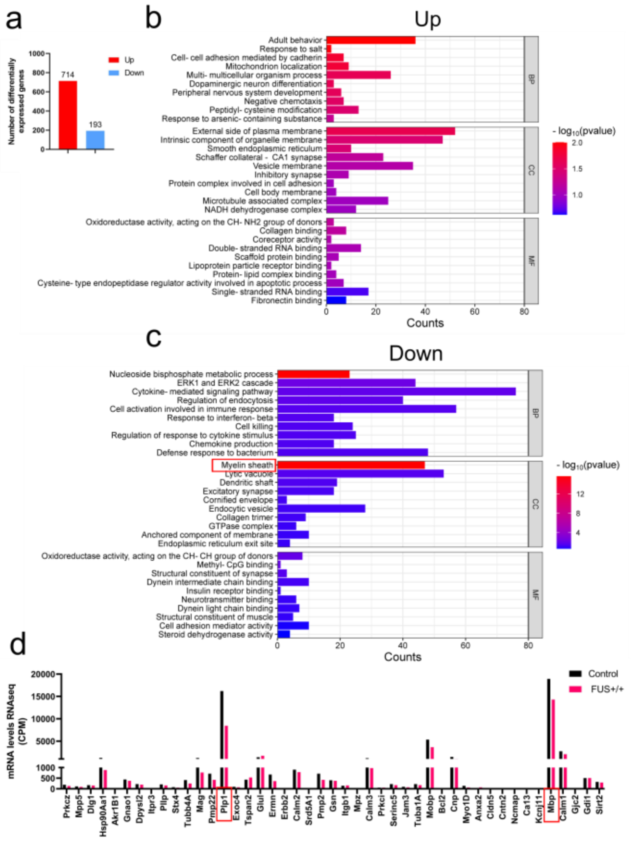

Supplementary Fig. 7 Transcriptome analysis showed myelin sheath signature in the spinal cord from symptomatic *FUS*$^{+/+}$ mice, compared to wild-type.

The RNAseq study performed by Rossaert and collaborators on the spinal cord of mice overexpressing wild-type human *FUS* under the prion promotor was reanalyzed [7]. **a** Number of DEGs (Fold-change>2, FDR<0.1) in the spinal cord from *FUS*$^{+/+}$ mice, compared to wild-type. **b, c** Bar plot displaying the 10 gene sets most significantly enriched among the up-regulated (**b**) and the down-regulated (**c**) pathways in the spinal cord of *FUS*$^{+/+}$ mice compared to wild-type, based on GSEA (minGSSize = 5; maxGSSize = 2000). **d** Normalized expression of myelin sheath-related genes in the spinal cord of *FUS*$^{+/+}$ mice, compared to wild-type, based on CPM from RNAseq.

Supplementary Fig. 8

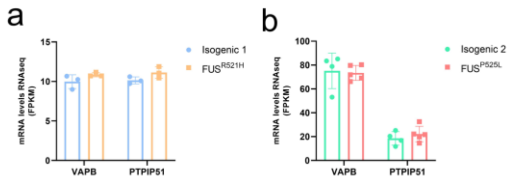

Supplementary Fig. 8 The mRNA levels of *VAPB* and *PTPIP51* were not different in mutant *FUS* OPCs compared to their isogenic controls.

a, b Normalized expression of *VAPB* and *PTPIP51* in *FUS*^{R521H} mutant **(a)** and *FUS*^{P525L} mutant **(b)**, compared to their respective isogenic control OPCs based on FPKM from RNAseq (*N*≥3 independent differentiations). Statistical analyses were performed by unpaired two-tailed *t*-tests to compare mutant *FUS* OPCs and their controls. Data are represented as mean ± SD.

Supplementary Fig. 9

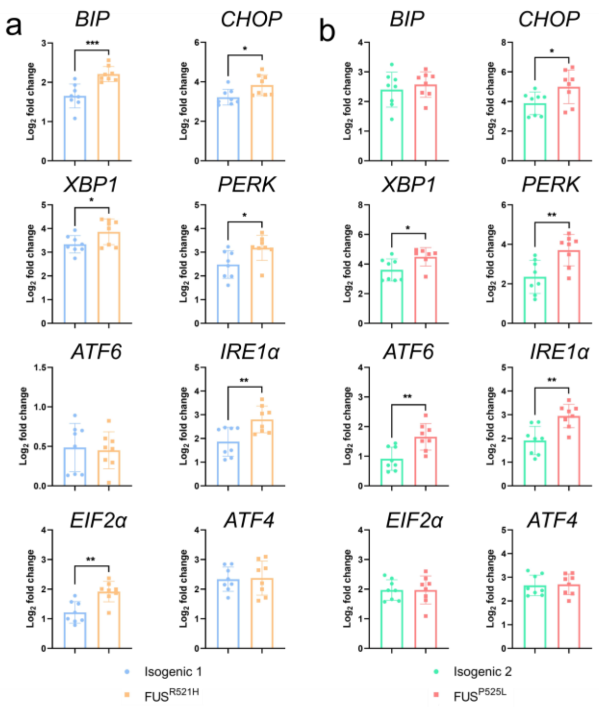

Supplementary Fig. 9 Mutant *FUS* OPCs show enhanced ER stress after thapsigargin exposure.

a, b RT-qPCR of *BIP*, *CHOP*, *XBP1*, *PERK*, *ATF6*, *IRE1α, eIF2α,* and *ATF4* transcripts after 2 µM thapsigargin exposure for 4 h in FUS^{R521H} mutant and isogenic control **(a)** and FUS^{P525L} mutant and isogenic control OPCs **(d)** ($N \geq 8$). Statistical analyses were performed by unpaired two-tailed *t*-tests to compare mutant *FUS* OPCs and their respective controls. Data are represented as mean ± SD. *$p<0.05$ **$p<0.01$.

Supplementary table 1. List of material and resources

REAGENT or RESOURCE	SOURCE	CAT
**Antibodies**		
Chicken anti-MBP (dilution 1:100)	Millipore	Cat# AB9348
Rabbit anti-FUS/TLS (dilution 1:100)	Proteintech	Cat#115701-AP
Mouse anti-O4 (dilution 1:200)	Bio-techne	Cat#MAB1326
Rabbit anti-BIP (dilution 1:1000)	Cell Signaling Technology	Cat#3183S
Mouse anti-CHOP (dilution 1:1000)	Cell Signaling Technology	Cat#2895
Rabbit anti-IRE1a (dilution 1:1000)	Cell Signaling Technology	Cat#3294
Rabbit anti-ATF4 (dilution 1:1000)	Cell Signaling Technology	Cat#11815S
Rabbit anti-β-actin (dilution 1:1000)	Cell Signaling Technology	Cat#8457S
Rabbit anti-PTPIP51 (dilution 1:400)	Abcam	Cat#ab224081
Mouse anti-VAP-B (dilution 1:400)	Bio-techne	Cat#MAB58551
Goat Anti-Mouse Immunoglobulins/HRP (dilution 1:2000)	Dako	Cat#P044701-2
Swine Anti-Rabbit Immunoglobulins/HRP (dilution 1:2000)	Dako	Cat#P021702-2
Rabbit anti-Rat IgG (H+L) Secondary Antibody, HRP (dilution 1:2000)	Thermo Fisher Scientific	Cat#61-9520
Goat anti-rabbit Alexa488 (dilution 1:1000)	Thermo Fisher Scientific	Cat#A11017
Donkey anti-mouse Alexa Fluor 555 (dilution 1:1000)	Thermo Fisher Scientific	Cat#A31570
Goat anti-Chicken IgY (H+L) Cross-Adsorbed Secondary Antibody, Alexa Fluor™ Plus 647 (dilution 1:1000)	Thermo Fisher Scientific	Cat#A32933
Hoechst 33342 Solution (dilution 1:2000)	Thermo Fisher Scientific	Cat#62249
CellMask™ Plasma Membrane Stains (dilution 1:1000)	Thermo Fisher Scientific	Cat#C10046
Conjugated O4-APC (dilution 1:100)	Miltenyi	Cat#130-115-810
ProLong™ Gold Antifade Mountant with DNA Stain DAPI	Thermo Fisher Scientific	Cat#P36931
**Chemicals, peptides, and recombinant proteins**		
Accutase™	Gibco™	Cat#A1110501
SB431542	Tocris Bioscience	Cat#1614
LDN-193189	Miltenyi	Cat#130-106-540
NT3 (Neurotrophin-3) Animal Free	Peprotech	Cat#AF-450-03
Recombinant Human HGF (insect-derived)	Peprotech	Cat#100-39
Retinoic acid	Sigma-Aldrich	Cat#R2625
Recombinant Human insulin-like growth factor (IGF)-1	Peprotech	Cat#100-11
T3 tri-iodo-L-thyronine	Sigma-Aldrich	Cat#T2877
db-cAMP	Sigma-Aldrich	Cat#D0627
smoothened agonist,SAG	Merck Millipore	Cat#566660
Recombinant Human PDGF-AA	Peprotech	Cat#100-13A
Biotin	Sigma-Aldrich	Cat#B4639
Insulin solution human	Sigma-Aldrich	Cat#I9278
Laminin from Engelbreth-Holm-Swarm murine sarcoma basement membrane	Sigma-Aldrich	Cat#L2020
Poly-L-ornithine hydrobromide	Sigma-Aldrich	Cat#P3655
Y-27632	Merck Millipore	Cat#688001
Doxycycline hyclate	Sigma-Aldrich	Cat#D9891
Hygromycin B	Gibco™	Cat#10687010
Puromycin dihydrochloride	Sigma-Aldrich	Cat#P8833
Fialuridine	Sigma-Aldrich	Cat#SML0632
N-2 Supplement (100X)	Gibco™	Cat#17502048
B-27™ Supplement, minus vitamin A (50X)	Gibco™	Cat#12587010
Revitacell (100X)	Gibco™	Cat#A2644501
MEM Non-Essential Amino Acids Solution (100X)	Gibco™	Cat#11140050
2-Mercaptoethanol	Gibco™	Cat#21985023

Penicillin-Streptomycin	Gibco™	Cat#15070063
Thapsigargin Epoxide	alomone labs	Cat#T-650
**Software and algorithms**		
NIS-Elements AR 4.30.02/5.40 Software	Nikon	N/A
Operetta High Content Imaging System	Revvity	N/A
Columbus	PerkinElmer Health Sciences	http://columbia.psb.ugent.be/
ImageJ 1.52p software	NIH	https://imagej.nih.gov/ij/
Prism 9.0.0	GraphPad	https://www.graphpad.com/scientific-software/prism/

Supplementary table 2. List of RT-qPCR primer sequences

Gene	Forward Primer	Reverse Primer
OCT4	GATGGCGTACTGTGGGCCC	TGGGACTCCTCCGGGTTTTG
NKX2.2	AAACCGTCCCAGCGTTAAT	CGGCTGACAATATCGCTACTC
OLIG1	GTACTCCTGCGTGTTAATGAGA	GCATCCAGTGTTCCCGATT
SOX10	CTTCATGGTGTGGGCTCAGG	CACTTTCGTTCAGCAGCCTC
MBP	AGGATTTGGCTACGGAGGCAGA	GGTTTTCAGCGTCTAGCCATGG
MOG	GTAGCCAGTTGTAGCAGATGA	AACTTCGAGCAGAGATAGAGAATC
BIP	TGTTCAACCAATTATCAGCAAACTC	TTCTGCTGTATCCTCTTCACCAGT
CHOP	CAAACAGTCTATGCCACAAGT	AGCGACAGAGCCAAAATCAG
XBP1	CCCTCCAGAACATCTCCCCAT	ACATGACTGGGTCCAAGTTGT
PERK	ACGATGAGACAGAGTTGCGAC	ATCCAAGGCAGCAATTCTCCC
ATF6	TCCTCGGTCAGTGGACTCTTA	CTTGGGCTGAATTGAAGGTTTTG
eIF2α	CCGCTCTTGACAGTCCGAG	GCAGTAGTCCCTTGTTAGTGACA
IRE1α	CACAGTGACGCTTCCTGAAAC	GCCATCATTAGGATCTGGGAGA

Supplementary table 3. List of primers for Sanger sequencing

Name primer	Forward sequence	Reverse sequence	Off target sequence
FUS-P525 locus	CATTTTGAGGGCTAGGTGGA	AGTGAAAAGGGGGAAGAGGA	
Off target 1	GAACGTAGCTGGAACTGC	TGACACTGGAGCTATCAGC	AAATGCCCTAATAATTAGCC
Off target 2	TGCCATGTGAGAGAATCGC	AAAGCACTTCTTGTCTCCC	AGACACCTATTAATTAGCC
Off target 3	GGTTGCAGCTGAGACTGC	CAGCTAGCCTGGCTATCAGG	AAAGACCATAATAATTAGCC
Off target 4	CCTGACTCTGGTGTTTGCC	AGAATAGGCCCGCTCACC	AGAACCCATACTAATTAGCC
Off target 5	AGCCTGTCTTCTTTACGC	TCTTAGGTGTAGGCATTAGG	TGTGGCTGTTTTAATTAGCC
Off target 6	AGTGTTGCTTGCCAAAGGC	TCTGTACACAAAGGGCATGG	AGAAGCCTTACTAATTAGCC
Off target 7	AACATTCAGCCCTGCTTCG	ACCTCCTGACCAAGTGC	AGCTGCAGTATTAATTAACC
Off target 8	GTTAGCCAGCTTTCAAGTGG	CTTCCAGGAGACACTTCCC	GGTGGCCCTATTAATTAGCG
Off target 9	GATGGCTGAGTATCTCATCC	GTAGCAAGTGATGATAAGACC	AGAGACAATATTAGTTAGCC

Supplementary table 4. List of RT-PCR primer sequences for intron retention

Name	Orientation	Target	Sequence (5’-3’)
hFUS-Exon 6-F1	Forward	FUS exon 6	TCCTCCATGAGTAGTGGTGGT
hFUS-Intron 6-R4	Reverse	FUS intron 6	GTTCAGGCTCCCAAGTTCTC
hFUS-Intron 7-F3	Forward	FUS intron 7	TTCTCTCGGGTGAGAGAACC
hFUS-Exon 8/9-R2	Reverse	FUS exons 8 and 9	GTCTGAATTATCCTGTTCGGAGTC

SUPPLEMENTAL EXPERIMENTAL PROCEDURES

Recombinase-mediated cassette exchange

Recombinase-mediated cassette exchange (RMCE) to integrate TET-ON-SOX10 in the AAVS1 locus was performed as described in Ordovas et al. [6] as well as Garcia et al. [1, 2]. In short, iPSCs were detached using accutase (Sigma), and single-cell suspensions (106 cells) were resuspended in 100 µl Nucleofector SolutionTM (Lonza) with 5 µg of a donor plasmid and 2 µg mRNA coding for the left and right ZFNs. The donor plasmid contains homology arms flanking the ZFN-mediated double-strand break with in between the homology arms a combination of GFP and hygromycin/thymidine kinase (TK) located between FRT and FRT3 sites. Following nucleofection with the Amaxa nucleofector device (program F16), single cells were plated on matrigel-coated plates in mTeSRTM1 medium (Stemcell Technologies) supplemented with rock inhibitor (Y-27632, VWR). When small colonies appeared, hygromycin (Hygromycin B, Gibco™) selection was performed by gradually increasing hygromycin concentrations from 50 to 300 µg/ml. Single colonies were manually picked and cultured separately. Quality control (QC) consisted of 5' junction PCR to ensure insertion inside the AAVS1 locus and southern blot to detect possible random integration events. hiPSC clones that passed all QC, were subsequently subjected to RMCE to replace the GFP-Hygro-TK cassette with a cassette containing FRT and FRT3 sites in identical orientation, a promotorless puromycin cassette for gene trapping and an inducible TET-ON system for overexpression of SOX10. Nucleoporation and subsequent selection were again performed as described by Ordovas et al. Correctly recombined cells were selected by initial positive selection using puromycin (Sigma; 120 to 300 ng/µl) followed by negative selection with 0.5 µM Fialuridine (FIAU, Sigma).

Base editing

To introduce the *P525L* mutation in WT-iSOX10 iPSCs, we used the base editing protocol described by Nami et al. [5]. Briefly, WT-iSOX10 iPSC colonies were detached with accutase and single-cell suspensions (106 cells) resuspended in 100 µl Nucleofector SolutionTM with 2.5 µg U6-sgRNA plasmid (5'- AGAGGCCGTATTAATTAGCC -3') and 7.5 µg of the base editor plasmid containing GFP and Cas9 nickase fused to cytidine deaminase (pCMV_AncBE4max_P2A_GFP, plasmid #112100 from Addgene). Following nucleofection with the Amaxa nucleofector device (program F16), single cells were plated on matrigel-coated plates in mTeSRTM1 medium supplemented with rock inhibitor. When small colonies appeared (after 2-3 days), GFP+ cells were sorted by FACS and plated on matrigel-coated plates in mTeSRTM1 medium supplemented with rock inhibitor. After 10-14 days, single colonies were manually picked and cultured separately. For each clone, the *FUS* gene was sequenced via Sanger sequencing and clones in which the base editing was successful, possible off-target events were investigated. Top 10 off-target regions were defined using IDT tool (https://eu.idtdna.com/site/order/designtool/index/CRISPR_SEQUENCE), and target regions were sequenced via Sanger sequencing. All primer sequences can be found in Supplementary Table 3.

Generation of iPSC-derived oligodendrocytes

OPCs were generated as previously described, with some modifications [1, 2]. Single-cell suspensions of iSOX10-iPSCs were plated at a density of 25 000 cells/cm2 on matrigel-coated plates in mTeSRTM1 medium supplemented with 1X Revitacell (Gibco). 48 hours later, the medium was changed to basal medium supplemented with 1 µM LDN-193189 (Miltenyi), 10 µM SB431542 (Tocris) and 100 nM RA (Sigma) with daily medium changes for 7 days. Basal medium consisted of DMEMF12 GlutamaxTM (Gibco) supplemented with 1X N2 supplement (Gibco), 1X B27 supplement without vitamin A (Gibco), 25 µg/ml human insulin solution (Sigma), 5 µM 2-Mercaptoethanol (Gibco), 1X MEM NEAA (Gibco) and 100 U/ml Penicillin-streptomycin (Gibco). For the next 4 days, basal medium supplemented with 100 nM RA and 1 µM SAG (Millipore) was added to the cells and changed daily. Next, DIV12 NPCs were detached using accutase, and single-cell suspensions were plated on

matrigel-coated plates at 20,000 cells/cm^2 in basal medium supplemented with 100 nM RA, 1 µM SAG and 1X Revitacell. The following day, the medium was changed every other day.

RT-PCR intron retention validation

For human FUS, the forward primer was designed for exon 6 and the reverse primer designed to span the spliced exon 8/9 junction to preferentially amplify spliced FUS mRNA. An additional third primer was designed to amplify a section of either intron 6 or intron 7 [3]. Primer sequences are listed in Supplementary Tables 4. RNA was isolated with the Quick-RNA Microprep Kit (Zymo research) according to the manufacturer's instructions. cDNA was obtained from extracted RNA using SuperScript™ IV VILO™ Master Mix with ezDNase enzyme (Thermofisher), according to the manufacturer's instructions.

RT-PCR was carried out using Q5® High-Fidelity 2X Master Mix (New England Biolabs). Each PCR reaction mix contained 5 ng of cDNA, 10 mM of forward and reverse primers. cDNA was amplified with the following conditions: Intron 6/7 retention: One cycle of 30s at 98◦C, followed by 30 cycles of 10 s at 98◦C, 30 s at 67◦C and 30 s at 72◦C, and finishing with 2 min incubation at 72◦C. 10 µL of the PCR products were loaded on a 2 % agarose (Thermo Scientific) gel electrophoresis with 1 Kb Plus DNA Ladder (Thermo Scientific). For quantification, we quantified individually the signal intensities of the two bands, and computed a % of intron retention as such: (intensity of Intron + band)/ (intensity of Intron + band + intensity of Intron- band)* 100.

SUPPLEMENTAL REFERENCES

1. García-León JA, García-Díaz B, Eggermont K, Cáceres-Palomo L, Neyrinck K, Madeiro da Costa R, Dávila JC, Baron-Van Evercooren A, Gutiérrez A, Verfaillie CM (2020) Generation of oligodendrocytes and establishment of an all-human myelinating platform from human pluripotent stem cells. Nat Protoc 15:3716–3744. doi: 10.1038/s41596-020-0395-4

2. García-León JA, Kumar M, Boon R, Chau D, One J, Wolfs E, Eggermont K, Berckmans P, Gunhanlar N, de Vrij F, Lendemeijer B, Pavie B, Corthout N, Kushner SA, Dávila JC, Lambrichts I, Hu W-S, Verfaillie CM (2018) SOX10 Single Transcription Factor-Based Fast and Efficient Generation of Oligodendrocytes from Human Pluripotent Stem Cells. Stem cell reports 10:655–672. doi: 10.1016/j.stemcr.2017.12.014

3. Humphrey J, Birsa N, Milioto C, McLaughlin M, Ule AM, Robaldo D, Eberle AB, Kräuchi R, Bentham M, Brown A-L, Jarvis S, Bodo C, Garone MG, Devoy A, Soraru G, Rosa A, Bozzoni I, Fisher EMC, Mühlemann O, Schiavo G, Ruepp M-D, Isaacs AM, Plagnol V, Fratta P (2020) FUS ALS-causative mutations impair FUS autoregulation and splicing factor networks through intron retention. Nucleic Acids Res 48:6889–6905. doi: 10.1093/nar/gkaa410

4. Li J, Jaiswal MK, Chien J-F, Kozlenkov A, Jung J, Zhou P, Gardashli M, Pregent LJ, Engelberg-Cook E, Dickson DW, Belzil V V, Mukamel EA, Dracheva S (2023) Divergent single cell transcriptome and epigenome alterations in ALS and FTD patients with C9orf72 mutation. Nat Commun 14:5714. doi: 10.1038/s41467-023-41033-y

5. Nami FA, Ramezankhani R, Vandenabeele M, Vervliet T, Vogels K, Urano F, Verfaillie C (2021) Fast and Efficient Generation of Induced Pluripotent Stem Cell Lines Using Adenine Base Editing. Cris J 4:502–518. doi: 10.1089/crispr.2021.0006

6. Ordovás L, Boon R, Pistoni M, Chen Y, Wolfs E, Guo W, Sambathkumar R, Bobis-Wozowicz S, Helsen N, Vanhove J, Berckmans P, Cai Q, Vanuytsel K, Eggermont K, Vanslembrouck V, Schmidt BZ, Raitano S, Van Den Bosch L, Nahmias Y, Cathomen T, Struys T, Verfaillie CM (2015) Efficient recombinase-mediated cassette exchange in hPSCs to study the hepatocyte lineage reveals AAVS1 locus-mediated transgene inhibition. Stem Cell Reports 5:918–931. doi: 10.1016/j.stemcr.2015.09.004

7. Rossaert E, Pollari E, Jaspers T, Van Helleputte L, Jarpe M, Van Damme P, De Bock K, Moisse M, Van Den Bosch L (2019) Restoration of histone acetylation ameliorates disease and metabolic abnormalities in a FUS mouse model. Acta Neuropathol Commun 7:107. doi: 10.1186/s40478-019-0750-2

8. Tam OH, Rozhkov N V, Shaw R, Kim D, Hubbard I, Fennessey S, Propp N, Fagegaltier D, Harris BT, Ostrow LW, Phatnani H, Ravits J, Dubnau J, Gale Hammell M (2019) Postmortem Cortex Samples Identify Distinct Molecular Subtypes of ALS: Retrotransposon Activation, Oxidative Stress, and Activated Glia. Cell Rep 29:1164-1177.e5. doi: 10.1016/j.celrep.2019.09.066
